# Genotype-by-sequencing–enabled genome-wide association studies reveal genetic architecture of biomass and nitrogen modulation in tepary bean (*Phaseolus acutifolius*)

**DOI:** 10.1093/g3journal/jkag119

**Published:** 2026-05-05

**Authors:** Sri Kiran Reddy Alla, Vijay Joshi

**Affiliations:** Department of Horticultural Sciences, Texas A&M University, College Station, TX 77843, United States; Texas A&M AgriLife Research and Extension Center, Uvalde, TX 78801, United States; Department of Horticultural Sciences, Texas A&M University, College Station, TX 77843, United States; Texas A&M AgriLife Research and Extension Center, Uvalde, TX 78801, United States

**Keywords:** genome wide association studies, genotyping-by-sequencing, nitrogen uptake, genetic loci, candidate genes, marker-assisted selection, introgression breeding

## Abstract

*Phaseolus acutifolius* (tepary bean) is a heat- and drought-tolerant legume adapted to semiarid environments with emerging genomic resources, yet the genetic architecture of biomass and nitrogen-related traits remains poorly resolved. Here, we aimed to resolve the genetic architecture of cover-crop–relevant traits in tepary bean, anticipating predominantly polygenic control but allowing for a major-effect component of flowering time. We assessed 206 accessions and 4 commercial checks for biomass, flowering time, leaf amino acids, and a relative NUE-Index that integrates plant N uptake with seasonal soil N changes. Genotyping-by-sequencing produced 49,384 high-quality SNPs for multimodel genome-wide association studies. The results show substantial natural variation across traits, enabling association mapping. Biomass-associated loci on chromosomes 6, 7, and 11 align with candidate genes involved in structural growth and development, including hydroxyproline-rich glycoproteins and carotenoid cleavage dioxygenase 1. Relative NUE-Index loci implicate trehalose-6-phosphate signaling and phosphatase activity, supporting a role for coordinated carbon–nitrogen regulation in nitrogen uptake efficiency-related physiology. Leaf ureide and amino acid profiles showed pronounced among-accession variation, providing a complementary physiological context for nitrogen uptake efficiency-related trait variation. A major QTL on chromosome 3 for flowering time, near a BTB-domain gene, highlights a candidate region for phenology tuning in tepary bean. Overall, SNP data from genotyping-by-sequencing and genome-wide association studies reveal a largely distinct polygenic structure across traits in tepary bean, providing actionable loci and hypotheses for marker-assisted breeding and introgression toward resilient summer cover crop varieties.

**List of abbreviations**
GWAS: Genome-wide association studies
NUE: Nitrogen uptake efficiency
BLUPs: Best linear unbiased predictions
BLUEs: Best linear unbiased estimates
GBS: Genotype-by-sequencing
SNP: Single nucleotide polymorphism
DTF: Days to flower
VCF: Variant call format
GAPIT: Genome Association and Prediction Integrated Tool

## Introduction

Exploring native crops and identifying new genetic sources are imperative for addressing environmental stresses and developing resilient species for sustainable agriculture. Tepary beans (*Phaseolus acutifolius* A. Gray) are indigenous to northern Mexico and the southwestern United States (Moghaddam et al. 2021). Many cultivated grain legumes are sensitive to heat and drought stress, but tepary bean is recognized as more tolerant, making it a valuable genetic resource for warm, water-limited production systems (Ravelombola et al. 2024). These beans were domesticated and traditionally cultivated by indigenous communities in the southwestern United States; however, their usage declined due to their small seed size and restricted market demand (Moghaddam et al. 2021). Given their resilience to heat and drought, tepary beans may serve as a sustainable crop amid environmental changes (López-Hernández et al. 2025). They resemble common dry beans but are slightly smaller and rich in protein and essential minerals vital to human health (Porch et al. 2017). They can flourish under extreme temperatures with minimal irrigation, conditions that threaten legumes such as common beans and soybeans (Buitrago-Bitar et al. 2021; Moghaddam et al. 2021). Furthermore, tepary beans can form root nodules with specific *Bradyrhizobium* strains, facilitating atmospheric nitrogen fixation through symbiosis (Mohrmann et al. 2017). Despite their favorable traits, tepary beans remain under-researched and underutilized within agriculture. Recent genetic studies have enhanced agronomic characteristics, resulting in new cultivars with larger seeds and improved disease resistance (Porch et al. 2024).

One notable advantage of tepary beans is their potential utility as a cover crop within sustainable farming systems. Cover crops are cultivated between main crops to prevent soil erosion, suppress weeds, recycle nutrients, and promote soil health. Many traditional cover crops are unable to withstand summer heat and drought, restricting available options (Yang et al. 2023). Since tepary beans are native to hot, arid environments and have nitrogen-fixing capabilities, they could serve as effective summer cover crops, supporting soil health and sustainable, low-input agriculture. Their physiological resilience and nitrogen-fixing capacity position them as particularly suitable for regions where conventional cover crops fail under high vapor pressure and soil heat (Mohrmann et al. 2017; Yang et al. 2023). Adapted to arid and semiarid environments, tepary beans' stress tolerance underscores their promise for sustainable production and breeding initiatives focused on heat- and drought-resistance (Mhlaba et al. 2018). Traits such as biomass production, nitrogen uptake efficiency, and leaf amino acid content serve as key indicators of their effectiveness as cover crops. Increased biomass enhances ground cover, weed suppression, and soil carbon sequestration (Koudahe et al. 2022).

Nitrogen uptake efficiency is defined as the plant's capacity to absorb soil nitrogen to support biomass growth. Additionally, the analysis of leaf amino acids in various tepary bean germplasm concentrates on nitrogen-rich compounds such as asparagine, arginine, and allantoin, which are vital for nitrogen transport and storage within legumes (Lescano 2020). Investigating these profiles can elucidate the genetic potential for nitrogen accumulation. Although genome-wide studies in legumes like soybean have identified loci associated with nitrogen metabolism (Dhanapal et al. 2015), comparable research in tepary beans remains limited. Allantoin and other ureides are principal long-distance transport forms of fixed nitrogen, linking ureide levels in leaves with nitrogen remobilization efficiency (Smith and Atkins 2002; Werner et al. 2010). Transporters such as amino acid permeases and ureide permeases regulate their movement (Rentsch et al. 2007; Tegeder and Rentsch 2010), providing a mechanism to assess nitrogen physiology via these metabolites. Advances in genetic research, including genome-wide association studies (GWAS), which analyze single-nucleotide polymorphism (SNP) variation across numerous accessions, have enhanced understanding of complex plant traits (Chan et al. 2010). High-throughput sequencing techniques, such as genotype-by-sequencing (GBS), facilitate rapid identification of trait-associated genetic loci (Li et al. 2010). Traits pertinent to the performance of tepary beans as summer cover crops—such as biomass, flowering time, leaf amino acid content, and nitrogen uptake efficiency—are polygenic and influenced by environmental and physiological variables (Chan et al. 2010; Dhanapal et al. 2015). GWAS approaches can resolve these trait networks, especially given the recent expansion of tepary bean genomic resources (Mhlaba et al. 2018; Bornowski et al. 2023; Ravelombola et al. 2024). The recent availability of a tepary bean reference genome (Moghaddam et al. 2021) indicates that breeding initiatives remain in their early stages. Past genomic research has focused on seed yield, seed weight, and disease resistance (Bornowski et al. 2023; Ravelombola et al. 2024), identifying genetic loci and candidate genes for cultivar enhancement. Nevertheless, traits related to cover crop performance warrant further investigation.

Accordingly, we assessed a diverse collection of tepary bean accessions for biomass, flowering time, leaf amino acids—including allantoin, asparagine, and arginine—and a relative nitrogen-use efficiency index that balances nitrogen uptake with soil nitrogen dynamics (Seo and Kim 2020). Based on quantitative genetics, we hypothesized that most complex agronomic and metabolic traits are polygenic, with multiple small-to-moderate-effect loci, while key phenological traits, such as days to flowering, may also be influenced by one or a few major-effect loci associated with developmental phase transitions and environmental cue integration. Using multimodel GWAS, we identified significant SNP–trait associations and candidate genes underlying these physiological and agronomic traits, thereby providing crucial genomic insights to facilitate introgression breeding of tepary genotypes tailored for summer cover cropping and to improve *Phaseolus* crops.

## Materials and methods

### Germplasm and experimental site

A total of 206 *P. acutifolius* accessions and 4 commercial tepary checks (Sacaton Brown, Sonoran White, Blue Speckled, Black) were evaluated at the Texas A&M AgriLife Research and Extension Center (Uvalde, TX) on a certified organic field. Accession identifiers and passport information were obtained from the USDA-ARS National Plant Germplasm System (NPGS) GRIN-Global database (Supplementary Table 1). Field management used standard organic practices, with drip irrigation applied on 7 dates between 2025 April 30 and 2025 July 7, to deliver a seasonal total of 130 mm, triggered by visual plant stress symptoms and physically observing soil moisture at the surface and subsurface levels with a hand shovel. The total in-season rainfall at the experimental plot during the experiment was 191 mm (Garner Field [KUVA] weather station at the Garner Field Airport in Uvalde, Texas). The field experiment was arranged as an augmented randomized complete block design (augRCBD) with 5 blocks; 4 commercial check varieties were replicated in each block, whereas the diversity panel accessions were unreplicated. Screening large diversity panels with limited seed and field capacity favored augmented layouts that maximize the number of genotypes observed while still providing replicated checks to estimate heterogeneity and sampling error (Williams et al. 2011; Federer and Crossa 2012; Möhring et al. 2014). Replicated checks distributed across blocks enable local control of field trends and the derivation of adjusted accession means, improving the reliability of genotype comparisons in early-stage trials (Smith et al. 2001; Piepho et al. 2012). For genomic analyses, augmented designs perform well within 2-stage pipelines, where stage-1 models account for blocks (and optional spatial trends) to produce BLUPs, and stage-2 leverages those adjusted means in association models (Williams et al. 2011; Federer and Crossa 2012, Damesa et al. 2019; Fernández-González and Isidro y Sánchez 2025).

### Phenotyping

Days to flower (DTF): DTF for each accession were recorded as the number of days from sowing until at least 50% of the plants in a plot have reached the flowering stage.

Biomass: For biomass measurements, plant accessions were harvested in triplicate, fresh biomass was determined, and the material was then dried at 70 °C for 5 to 6 d to determine dry biomass.

Relative NUE-Index: After measuring dry biomass, the same samples were analyzed for total N (TKN), NO_3_^−^, and NH_4_^+^ both presowing (PS) and after harvest (AH) using an EasyChem Plus analyzer (Chinchilla Scientific, Oak Brook, IL, United States) according to established protocols (Ketterings et al. 2017). We defined a relative NUE-Index to reflect the balance between plant N accumulation and seasonal depletion of extractable mineral N:

Plant N uptake = Dry biomass (g) × TKN (%)/100

Soil N_(PS or AH)_ = NO_3_-N + NH_4_^+^-N

Relative NUE-Index = Plant N uptake/Soil N removed

The net reduction in soil mineral N across the season was calculated as:

Soil N removed = Soil N_PS_ − Soil N_AH_.

Since legumes can combine soil N uptake with biological nitrogen fixation and access to mineralizable N pools, plant N accumulation might surpass the observed decrease in soil mineral N. Therefore, higher index values indicate greater N acquisition and use than seasonal depletion of soil mineral N under the tested conditions (Seo and Kim 2020). As the nodulation and fixation were not directly measured, the index is treated as an integrated performance metric rather than a direct proxy for nodulation traits. This functional index is appropriate for augmented field screens because it is computed per plot and does not require plot replication beyond the replicated checks used to stabilize stage-1 model error.

### Leaf amino acid extraction and analysis

Leaf amino acids were analyzed using established protocols (Joshi et al. 2019). Briefly, at flowering, fully expanded leaves were sampled, immediately chilled, freeze-dried, and ground with stainless steel balls (Abbott Ball Co., West Hartford, CT, United States) in a Harbil 5G-HD shaker. Approximately 16 to 18 mg of powder was extracted and derivatized using AccQ.Tag 3X Ultra-Fluor kit (Walters Corp., Milford, MA, United States), then quantified using a Waters Acquity H-class UPLC system equipped with a Waters Xevo TQ mass spectrometer with an electrospray ionization probe, which was used to detect individual amino acids. IntelliStart software (Waters Corp., Milford, MA, United States) was used to optimize each amino acid multiple reaction monitoring transition, collision energy values, and cone voltage. Instrument monitoring and data acquisition were performed using Waters’ MassLynx software. Data integration, calibration curves, and amino acid quantification were performed using the TargetLynx application manager. We focused on N-rich compounds (eg allantoin, asparagine, arginine) that are involved in transport and storage functions in legumes (Smith and Atkins 2002; Rentsch et al. 2007; Tegeder and Rentsch 2010; Werner et al. 2010).

### DNA extraction, GBS library construction, and SNP calling

DNA was extracted from young tepary bean leaves after 3 to 4 weeks of germination. DNA was extracted from leaf tissues using Qiagen DNeasy Plant Mini Kit following the manufacturer's protocol. Extracted DNA was measured using the DeNovix DS-11 Series Spectrophotometer. The quality of the DNA extracts was assessed based on concentration (ng/µL) and the 260/280 ratio (1.8 to 1.9). Pure DNA extracts were sent to the University of Minnesota Genomics Center (UMGC) (https://genomics.umn.edu/) for library preparation and sequencing. GBS libraries were prepared using *ApeKI* and sequenced as single-end 150 bp reads. Raw FASTQ quality was assessed with FastQC. Reads were trimmed to remove 5′ padding sequences and read-through into 3′ padding/adapters using GBStrim.pl, and trimmed reads were aligned to the *P. acutifolius* v1.0 reference genome with BWA-MEM. Alignment files were sorted and indexed with SAMtools, and alignment rates were summarized using Picard. Variants were called jointly across samples with FreeBayes using the parameters –use-best-n-alleles 4 –min-coverage 420 –limit-coverage 500, and the raw VCF was filtered with vcffilter to retain variants with QUAL > 20. The UMGC pipeline generated a filtered VCF by excluding variants genotyped in <95% of samples, variants with MAF < 1%, and samples with >50% missing genotypes. For downstream GWAS, we applied additional filtering prior to imputation to retain SNPs with MAF ≥ 0.05, site missingness ≤ 20%, and heterozygosity ≤ 10%. Missing genotypes were imputed using LD-kNNi (linkage disequilibrium k-nearest neighbor imputation) implemented in TASSEL. After filtering and LD-kNNi imputation, 49,384 high-quality SNPs were retained for population structure analysis and GWAS.

### Population structure analysis

Population structure analysis was performed on Structure 2.3.4. To reduce marker redundancy due to linkage disequilibrium (LD), SNPs were LD-pruned in PLINK using a sliding window of 50 SNPs and an LD threshold of *r*^2^ > 0.1. This pruning criterion was chosen to minimize redundancy among closely linked markers and to approximate a largely independent marker set for robust STRUCTURE and PCA inference. After LD pruning, 2,869 high-quality, nonredundant SNPs were retained for population structure analyses.

To estimate the number of subpopulations (*K*), STRUCTURE was run for *K* = 1 to 10 with 10 replicate runs per *K*. Each run used a burn-in of 50,000 iterations followed by 50,000 MCMC iterations. Delta *K* was evaluated using Structure Harvester (Pritchard et al. 2000), from which the optimal *K* was selected (Evanno et al. 2005). In this panel, the Evanno Δ*K* criterion showed a clear maximum at *K* = 2; therefore, *K* = 2 was used for the final STRUCTURE Q-matrix and bar plot. A *Q* matrix was generated, and a structure bar plot was established using the sort by Q option. The resulting *Q*-matrix was used to generate ancestry bar plots, with individuals sorted by membership coefficients (*Q*). In parallel, principal component analysis (PCA) was performed on genome-wide SNPs to obtain principal components and eigenvalues. The first 5 PCs were used as covariates to control for population structure in the GWAS (PCA = 5), and PCA plots were generated to visualize genetic differentiation.

### Statistical analysis

All analyses were conducted in R (version 4.3.3). GWAS were performed using the GAPIT R package (sourced from the ZZlab repository http://zzlab.net/GAPIT/gapit_functions.txt), and LD pruning and related population-genetic operations were conducted using PLINK (version 1.9). The analysis employed a 2-stage approach. In stage 1, mixed models estimated adjusted genotype values (BLUPs) along with their estimation error variance–covariance (EEV), capturing uncertainty associated with the augmented RCBD and the use of replicated checks. These adjusted means, BLUPs, and their EEV were then used as phenotypic inputs in marker models (Schulz-Streeck et al. 2013; Damesa et al. 2019; Fernández-González and Isidro y Sánchez 2025). The stage-1 mixed models adjusted phenotypes using the 5 blocks of the augmented RCBD; no additional spatial adjustment was applied. The adjustment model was of the form *y* = *μ* + Block + Genotype + *ε*, where Block captured the 5 blocks of the design; Genotype was treated as random to obtain BLUPs when estimable and as fixed when the genetic variance component collapsed. During stage 2, GWAS analyses in GAPIT used the stage-1 BLUPs as response phenotypes. Because GAPIT does not accept an EEV matrix as observation-level weights, the primary GWAS scans treated the adjusted values as point estimates (unweighted stage-2). The stage-wise framework is based on the idea that incorporating stage-1 uncertainty via EEV can enhance inference, especially for augmented designs (Damesa et al. 2019; Endelman 2023; Fernández-González and Isidro y Sánchez 2025). We used the adjusted phenotypes (BLUPs) for analysis, and sensitivity tests with alternative specifications confirmed the robustness of significant findings across various GWAS frameworks (Möhring et al. 2014; Fernández-González and Isidro y Sánchez 2025).

Linear mixed models for augmented RCBD: For each trait and environment, we fit linear mixed models appropriate for augmented RCBD to obtain genotype effects adjusted for blocks and checks. Checks and/or genotypes were treated as random to extract BLUPs when genotype variance was estimable; and for traits where the estimated genetic variance collapsed (eg due to limited replication in the augmented layout), we treated genotypes as fixed to obtain BLUEs, as is standard practice (Piepho et al. 2012; Schulz-Streeck et al. 2013). This first stage provides adjusted entry values that mitigate block-level heterogeneity and leverage check replication—core strengths of augmented designs (Smith et al. 2001; Williams et al. 2011; Federer and Crossa 2012; Möhring et al. 2014).

GWAS: The filtered VCF file was converted to a numeric format for GWAS. GWAS were performed using GAPIT (R) with GLM, MLM, BLINK, and FARMCPU models. Marker–trait associations were tested using a mixed linear model (MLM) of the form: *y* = *Xβ* + *sα* + *Zu* + *ε*, where *y* is the vector of phenotypes (stage-1 BLUPs), *X* is the design matrix for fixed covariates (including the intercept and population structure covariates such as PCs), *β* is the corresponding fixed-effect vector, *s* is the SNP genotype vector coded additively, *α* is the SNP effect, *Z* is the incidence matrix relating observations to random genetic effects *u*, and *ε* is the residual error. Population structure was controlled by PCs (*n* = 5) and, where applicable, by relatedness (*K*). We used a Bonferroni correction (*α* = 0.05/marker) to declare genome-wide significance (López-Hernández and Cortés 2019). For our current panel of 206 accessions and 49,384 high-quality markers, the Bonferroni-adjusted threshold is *P* ≤ 0.05/49,384 = 1.012 × 10^−6^, corresponding to −log10(*P*) ≥ 5.995 (reported as ∼6). To provide a less conservative, discovery-oriented assessment and to facilitate interpretation across traits, we also report Benjamini–Hochberg false discovery rate (FDR)–adjusted *P*-values (*q*-values; H&B.P.Value). Associations were classified as genome-wide significant when they exceeded the Bonferroni threshold and as FDR-significant when *q* < 0.05; signals below these thresholds are described as suggestive.

### LD decay and candidate-gene discovery

Genome-wide LD decay was assessed using the same SNP dataset used for GWAS by calculating pairwise *r*^2^ values for marker pairs within chromosomes and summarizing them as a function of physical distance. The LD decayed to *r*^2^ = 0.2 at roughly 14 kb (Supplementary Fig. 1). Therefore, we adopted a conservative ±10 kb window around each lead SNP to identify the most likely candidate genes in LD with the association signal. This window is slightly narrower than the overall LD decay distance, reducing the chance of including distant genes with weaker LD and minimizing false-positive candidate identifications. Consequently, candidate genes are considered as proximity-based hypotheses. Because LD varies across genomic regions, the ±10 kb window is used as a consistent, conservative rule for hypothesis generation rather than as proof of causality. For each significant SNP, we queried ±10 kb windows in Phytozome v14 to extract gene models and annotations, and compiled candidate genes for the associated traits (https://phytozome-next.jgi.doe.gov/).

## Results

### Phenotypic variations across the tepary bean panel

**Fig. 1.**
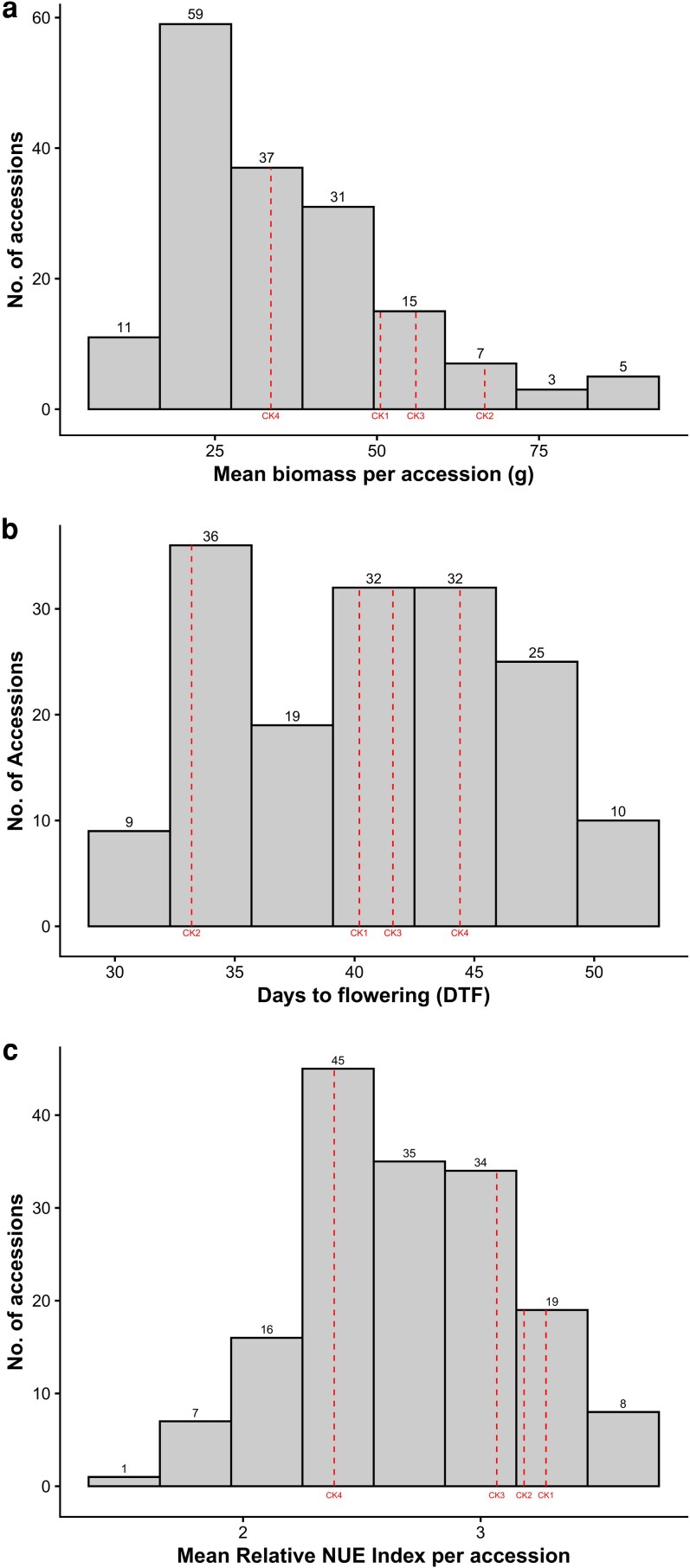
Phenotypic distributions of biomass, flowering time, and relative NUE-Index in *Phaseolus acutifolius*. a) Distribution of dry biomass (g plant^−1^) across 206 tepary bean accessions and 4 commercial checks. b) Distribution of days to flower (DTF). c) Distribution of relative NUE-Index values. For all panels, the *x*-axis represents phenotypic measurements, and the *y*-axis shows the number of accessions. Commercial check varieties are indicated with dashed reference lines.

We evaluated a diversity panel of tepary bean accessions from the USDA–NPGS to capture genetic variation, phenotyping DTF, above-ground biomass, leaf free amino acids, and a relative NUE-Index, covering phenology, productivity, and nitrogen-related physiology. In parallel, GBS produced a high-quality SNP dataset (49,384 markers after filtering and imputation), which was utilized for population structure analysis, GWAS, and downstream candidate gene studies. A wide range of phenotypic diversity was seen among the 206 tepary bean accessions and 4 commercial checks. Dry biomass varied from 12 to 90 g per plant, with check varieties averaging 51 g per plant (Fig. 1a). Several accessions, such as PI 666350, PI 692269, PI 549447, and PI 653254, showed notably high biomass, each producing between 70 and 93 g per plant. The traits DTF and the relative NUE-Index also displayed broad, continuous distributions typical of quantitative traits (Fig. 1b and c). Downstream analyses used adjusted genotypic values from stage-1 mixed models: BLUPs for biomass and NUE, and BLUEs for DTF and specific amino acids. Complete adjusted means for all traits are available in Supplementary Tables 2 and 3. Supplementary Tables provide trait-specific adjusted values; the count of nonmissing entries differs by trait because some accessions have missing phenotypic observations (marked as NA) and are excluded from the stage-1 adjustment and subsequent GWAS for that trait.

### Leaf amino acid profiles and multivariate structure

**Fig. 2.**
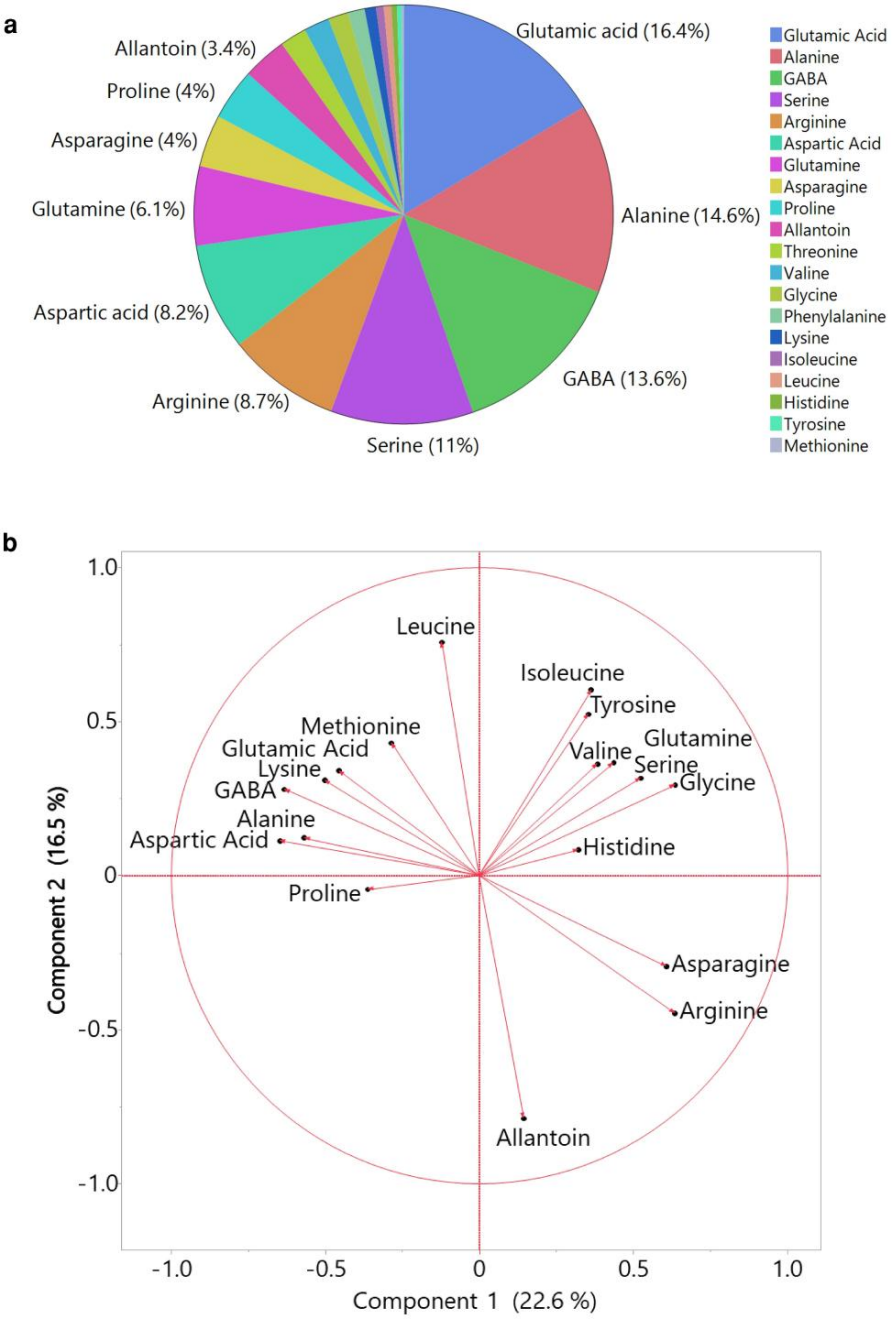
Leaf amino acid profiles and multivariate structure across tepary bean accessions. a) Proportional distribution (%) of 20 free amino acids in fully expanded leaves collected at flowering. b) PCA biplot of leaf amino-acid profiles. Arrows show feature loadings: direction indicates the association sign, and length shows the contribution. PC1 (22.6% variance) contrasts nitrogen-rich amino acids with negatively loaded amino acids. PC2 (16.5%) shows positive loadings for branched-chain amino acids and negative loadings for metabolites such as allantoin.

Twenty free amino acids were quantified across the germplasm panel. Nitrogen-rich compounds such as allantoin, asparagine, arginine, glutamic acid, and glutamine were consistently abundant but showed substantial among-accession variation (Fig. 2a; Supplementary Fig. 2). For example, the highest allantoin proportions (∼17.7% to 18.9%) occurred in W6 38698 and PI 653236, indicating pronounced genotypic differences in ureide-associated nitrogen transport/storage signatures.

PCA revealed a clear multivariate structure in the leaf amino acid dataset (Fig. 2b). We interpret the PCA using the biplot loading vectors: metabolites with vectors pointing in similar directions covary positively across accessions, vectors in opposite directions indicate negative covariance, and longer vectors indicate stronger contributions to the principal components. Along PC1 (22.6% of variance), asparagine and arginine load positively, whereas metabolites such as alanine and GABA load negatively, reflecting a dominant covariance axis contrasting nitrogen-allocation–related metabolites with metabolites more associated with central metabolism/stress-linked signatures. PC2 (16.5% of variance) is characterized by positive loadings for branched-chain amino acids (leucine, isoleucine, and valine), which co-load in similar directions (ie they covary rather than being differentiated from one another), and negative loadings for metabolites such as allantoin. Together, the vector groupings and contrasting orientations in Fig. 2b support coordinated covariance among subsets of amino acids while indicating multiple, partially decoupled biochemical axes that contribute to among-accession variation.

### SNP dataset generation, filtering, and quality control

GBS generated 830,410,742 single-end 150 bp reads across 210 samples (mean 3,954,336 reads per sample). Variant calling produced a raw VCF containing 210 samples, 719,967 markers, and 140,684 loci. UMGC pipeline-level filtering excluded variants genotyped in <95% of samples and variants with MAF <1% (and would remove samples with >50% missing genotypes; 0 samples were removed), yielding 140,936 markers on 43,380 loci. For downstream GWAS, we applied an additional GWAS-focused filtering step (MAF ≥ 0.05, site missingness ≤ 20%, and heterozygosity ≤ 10%) prior to imputation to retain high-quality SNPs for association testing. Missing genotypes were then imputed using LD-kNNi, yielding a final GWAS dataset comprising 49,384 SNPs.

### Population structure and genetic relatedness

**Fig. 3.**
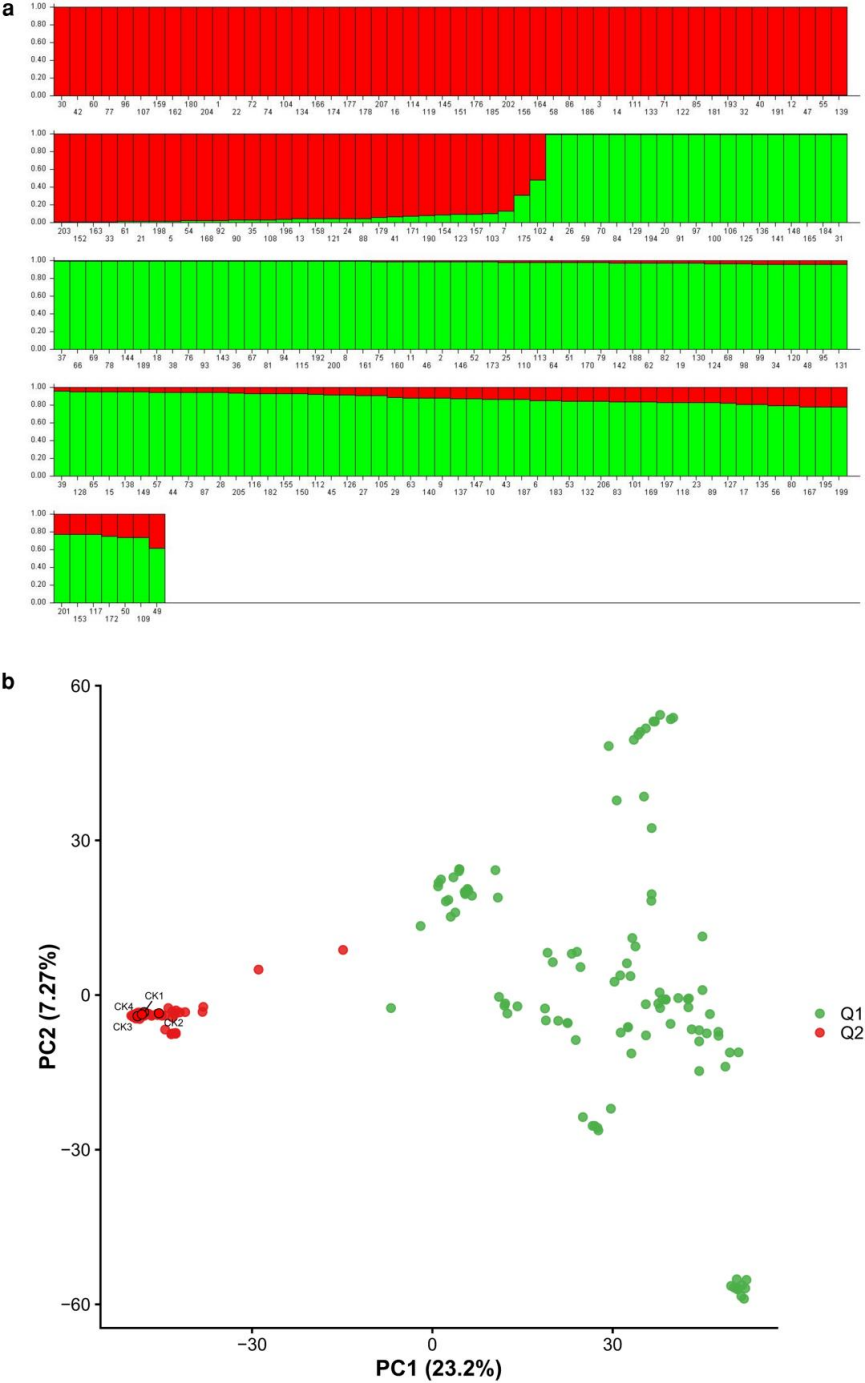
Population structure and genetic differentiation within the tepary bean diversity panel. a) STRUCTURE bar plot at *K* = 2 showing 2 subpopulations (Q1 = green, Q2 = red) across 206 accessions and 4 commercial checks. b) Principal coordinate analysis (PCoA) illustrating separation between commercial checks and the wider germplasm diversity. Delta-*K* values were estimated using Structure Harvester to determine the optimal number of clusters.

STRUCTURE and PCA analyses revealed 2 major subpopulations (*K* = 2) among the accessions (Fig. 3), consistent with the Evanno Δ*K* criterion. Approximately 120 accessions were grouped into Q1, and ∼80 accessions were grouped into Q2. All commercial check varieties clustered in Q2, suggesting a narrower genetic base among improved tepary types than among wild or landrace accessions. PCA corroborated these findings, with PC1 (23.2%) and PC2 (7.3%) distinguishing check varieties from the broader genetic diversity of the panel (Fig. 3b). This structure was controlled for in all GWAS models.

### Genome-wide associations and candidate gene analysis for biomass, days to flower, and relative NUE-Index

**Fig. 4.**
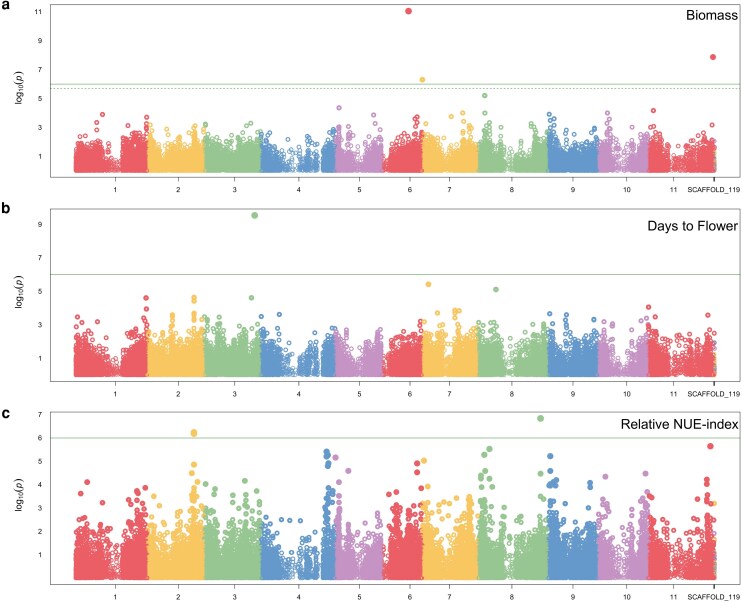
Genome-wide association results for biomass, flowering time, and relative NUE-Index. Manhattan plots display single-nucleotide polymorphism (SNP) associations across the 11 tepary bean chromosomes for: a) dry biomass, b) days to flower, and c) relative nitrogen uptake efficiency (relative NUE-Index). The *y*-axis shows –log_10_(*P*-value), and the genome-wide significance threshold (−log_10_*P* = 6) is indicated by the horizontal dashed line. Significant SNPs were used to identify downstream candidate genes.

GWAS using GLM, MLM, BLINK, and FarmCPU identified marker–trait associations for biomass, flowering time, and the relative NUE-Index (Fig. 4). For dry biomass, 3 loci exceeded the Bonferroni genome-wide threshold (−log_10_  *P* ≥ ∼6): S06_21574636 on Chr06 (*P* = 9.07 × 10^−12^), S11_51429071 on Chr11 (*P* = 1.36 × 10^−8^), and S07_135914 on Chr07 (*P* = 5.06 × 10^−7^). Candidate genes within the LD-supported window included Phacu.CVR.007G000700, encoding a hydroxyproline-rich glycoprotein implicated in cell wall structure, and Phacu.CVR.011G252000, encoding carotenoid cleavage dioxygenase 1 (CCD1) linked to growth and developmental processes. For DTF, the lead association (S03_40020268 on Chr03) exceeded the Bonferroni threshold (*P* = 2.96 × 10^−10^) and was also significant after FDR adjustment (H&B.P.Value = 1.46 × 10^−5^). This locus explained 54.9% of phenotypic variance in the BLINK analysis, indicating a major-effect component of flowering-time variation in this panel under the tested conditions. The lead SNP lies near Phacu.CVR.003G300600 (H-BTB1), a BTB domain protein annotated in the reference genome. For the relative NUE-Index, the strongest associations did not exceed the Bonferroni threshold but were significant after FDR correction (*q* < 0.05). Specifically, S08_49638740 (*P* = 1.47 × 10^−7^; H&B.P.Value = 0.00727) and S02_36911867 (*P* = 5.80 × 10^−7^; H&B.P.Value = 0.0109) mapped near Phacu.CVR.008G292300 (protein phosphatase 2C) and Phacu.CVR.002G277300 (trehalose-6-phosphate synthase/phosphatase), respectively. Collectively, these results highlight discrete genomic regions that influence biomass accumulation, phenological timing, and nitrogen-use-related physiology in tepary bean. Complete information for the lead QTLs for biomass, DTF, and the relative NUE-Index—including SNP identifiers, chromosome positions, raw and FDR-adjusted *P*-values, and all annotated gene models within the LD-supported ±10 kb interval—is provided in Supplementary Table 4.

### Genome-wide associations for leaf amino acids

**Fig. 5.**
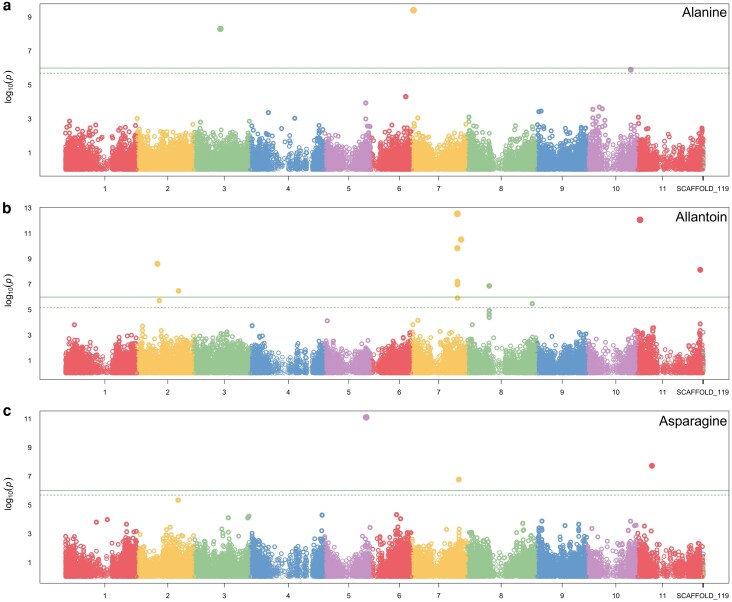
Genome-wide association results for key leaf amino acids. Representative Manhattan plots for 3 nitrogen-rich amino acids: a) alanine, b) allantoin, and c) asparagine. The *x*-axis displays the 11 chromosomes, and the *y*-axis shows –log_10_(*P*-value). SNPs surpassing the genome-wide significance threshold (−log_10_*P* = 6) were used for candidate gene discovery. Additional amino acid traits are presented in Supplementary Figs. 2 to 4.

Among the 20 quantified leaf amino acids, 14 traits showed significant SNP associations, highlighting widespread genetic variation in primary nitrogen-rich and carbon-rich metabolites. Each amino acid had between 1 and 7 significant loci, with example Manhattan plots for alanine, allantoin, and asparagine shown in Fig. 5. Allantoin, an important ureide involved in nitrogen transport and remobilization, had 7 significant associations, many of which were near genes linked to intracellular trafficking, chloroplast function, and vesicle-mediated transport. Both asparagine and arginine, crucial for nitrogen redistribution, exhibited multiple SNPs specific to certain traits but did not share loci with allantoin, indicating no pleiotropy despite their biochemical connection. Branched-chain amino acids, such as leucine, isoleucine, and valine, were associated with loci encoding metabolic regulators involved in protein turnover and biosynthesis. Notably, none of the amino acid traits shared SNPs or genomic regions, implying a highly polygenic and trait-specific genetic control of leaf metabolite levels in *P. acutifolius*. This aligns with previous findings in legumes, which show that metabolite profiles often segregate into independent biochemical modules (Fig. 5 and Supplementary Figs. 3 to 5).

### Candidate-gene identification and functional annotations of leaf amino acids

Candidate-gene exploration within ±10 kb of all genome-wide significant SNPs identified 111 unique genes with functions relevant to plant metabolism, nutrient transport, and developmental regulation. Functional annotations showed strong enrichment for gene families involved in nitrogen transport and allocation—such as components of the COPII vesicle-trafficking system, ureide transport proteins, and amino acid transporters—as well as regulators of intracellular signaling, including calcium-binding proteins and various kinase families. Genes related to primary metabolic pathways, such as trehalose-6-phosphate metabolism and multiple amino acid biosynthesis processes, were also identified. Several loci encoded structural cell-wall proteins, especially hydroxyproline-rich glycoproteins (HRGPs), highlighting their role in biomass accumulation in tepary bean. The lack of shared candidate genes across traits such as biomass, relative NUE-Index, flowering time, and amino acid levels underscores the independence of these physiological processes and suggests that tepary bean's genetic architecture for vegetative growth and nitrogen metabolism is highly partitioned. Supplementary Table 5 shows detailed information on significant SNPs, putative candidate genes, and functional annotations of leaf amino acids.

## Discussion

Across the evaluated tepary bean panel, GWAS identified trait-associated loci ranging from a major-effect signal for flowering time to more distributed patterns for physiological and metabolite traits. Consistent with our hypotheses, biomass and the relative NUE-Index were associated with multiple loci of moderate effect, while DTF was dominated by a major locus (S03_40020268) in this dataset. Leaf amino acid traits showed largely trait-specific association patterns, consistent with distributed genetic control and pathway-structured variation rather than extensive genome-wide pleiotropy. Overall, these findings lay a genetic foundation for improving nitrogen capture and related traits in tepary bean, while emphasizing that locus stability and effect sizes may vary across environments and genetic backgrounds. Tepary bean is known for its performance in hot, dry environments, and a key question in legumes is how biomass accumulation and allocation relate to flowering time under high temperature- and water-limited conditions. Although the present study did not impose controlled drought or heat treatments beyond ambient field conditions, our results suggest a broader framework in which earlier flowering may shorten vegetative duration and limit biomass, whereas later flowering may extend vegetative growth but increase risk under terminal stress. Future multienvironment trials exposing the same accessions to contrasting drought and heat regimes will be essential to test for conditional neutrality and guide variety development for summer production.

### Biomass candidate genes can serve as growth tools for tepary beans as a summer cover crop

Biomass accumulation is crucial for cover crops because increased vegetative growth enhances soil organic matter, prevents erosion, and suppresses weeds. Genes on chromosomes 6, 7, and 11 provide a direct genetic route for breeding tepary beans with quick ground cover and high plant residue—important traits for summer cover crops. Significant SNPs overlap with candidates like HRGP and CCD1, indicating a structural and developmental basis for growth, as HRGPs influence cell-wall flexibility and CCD1-derived apocarotenoids regulate growth (Johnson et al. 2017). The SNP on chromosome 11 within Phacu.CVR.011G252000, a CCD1 gene, suggests that variation in apocarotenoid metabolism may help the tepary bean produce robust biomass under stress. CCD1 controls carotenoid breakdown and produces signals that aid photoprotection, reactive oxygen species regulation, and stress adaptation—key for growth in drought and heat, where tepary bean performs well (Floss and Walter 2009; Dhar et al. 2020; Du et al. 2023). Lower CCD1 activity increases carotenoid and antioxidant levels, potentially boosting photosynthesis and biomass, thereby benefiting soil building (Du et al. 2023). Focusing on these genetic regions related to structure and growth is relevant for tepary bean, a crop suited to hot, dry climates and valued for its stress resistance (Mhlaba et al. 2018; Moghaddam et al. 2021). Higher biomass in cover crops accelerates canopy development, protects soil, and adds organic matter, thereby improving soil quality (Koudahe et al. 2022). The absence of overlap between biomass loci and those for flowering time or nitrogen-use efficiency suggests limited antagonistic pleiotropy, allowing breeders to combine growth traits on chromosomes 6, 7, and 11 with traits for flowering and nitrogen use without reducing residue benefits (Bornowski et al. 2023; Ravelombola et al. 2024). Overall, targeting regions near HRGP and CCD1 offers a feasible way to increase vegetative mass in tepary beans while maintaining other cover crop functions. Another gene in the same region, Phacu.CVR.011G252100 encodes SMC2, a component of the condensin complex required for proper meristem development and cell division. In Arabidopsis, SMC2 deficiency alters growth and cell division, suggesting that variants in tepary SMC2 could promote rapid early biomass and support rapid canopy closure (Siddiqui et al. 2003). The chromosome 7 biomass locus also includes a homeodomain-like gene, consistent with roles of HD-ZIP and KNOX transcription factors in organ formation, root–shoot balance, and adaptation (Ariel et al. 2007; Hay and Tsiantis 2010; Li et al. 2022). Nearby is an HRGP/extensin gene, important for strengthening and extending the cell wall-traits linked to stem strength and resistance to lodging, which improve field performance of cover crops (Marzol et al. 2018; Mishler-Elmore et al. 2021). These loci indicate that biomass in tepary bean involves stress responses, developmental control, and cell-wall architecture, supporting its resilience and soil-protective abilities as a summer cover crop.

### Relative NUE-Index and leaf amino acids reveal physiological differentiation in nitrogen fixation and mobilization

NUE is a key trait for cover crops, as it improves nitrogen cycling, reduces fertilizer needs, and enhances soil fertility. Two major SNP clusters include genes linked to carbon–nitrogen coordination, stress signaling, and transporter maturation. The gene Phacu.CVR.002G277300, involved in trehalose-6-phosphate (T6P) synthesis, plays a crucial role in sugar signaling via the T6P–SnRK1 system, which regulates carbon allocation and growth. Manipulating T6P has increased cereal yields and stress resilience by boosting source–sink carbon flow, implying that allelic variation in tepary bean T6P genes might influence nitrogen assimilation during cover-crop growth (Paul et al. 2018, 2020, 2022). Another gene, Phacu.CVR.008G292300 encodes a PP2C phosphatase involved in ABA signaling, which controls stomatal conductance, root development, and stress adaptationtraits that directly affect nitrogen uptake, especially under drought or heat conditions common in tepary cropping areas (Umezawa et al. 2010; Hsu et al. 2021). Nearby ALG6/ALG8 N-glycosylation genes contribute to protein folding and membrane trafficking, impacting the stability of nutrient transporters. Variations in this pathway may influence root nitrogen uptake and mobilization, which are vital ecological functions of cover crops (Strasser 2016; Dünser and Schoberer 2025). Collectively, the NUE-related genes suggest that tepary beans are well adapted to low-input systems, where efficient nutrient uptake and stress resilience promote soil nitrogen retention and cycling.

Leaf amino acid content reflects nitrogen assimilation, stress response, and metabolic readiness, all crucial for cover crop performance. Several loci linked to alanine, asparagine, and glutamate are associated with genes involved in organelle development, stress metabolism, and nitrogen remobilization. For instance, loci for alanine include TPR-domain proteins (Phacu.CVR.007G021700) and GHMP kinases. TPR proteins influence chloroplast development, tetrapyrrole biosynthesis, and chaperone activity, affecting photosynthesis and amino acid synthesis under stress conditions such as heat or drought (Hu et al. 2014; Ma et al. 2017; Herbst et al. 2024). These mechanisms support tepary bean's resilience and its ability to sustain amino acid production under environmental stress.

Regions associated with asparagine and glutamate overlap with genes involved in allantoin metabolism, particularly ureide transport and purine breakdown, which support stress tolerance and nitrogen recycling (Werner and Witte 2011; Lescano et al. 2020; Kaur et al. 2021). Allantoin activates ABA signaling, ROS defenses, and nitrogen remobilization, helping maintain amino acid pools during stressful periods—an important trait for a cover crop's stability and nitrogen cycling. The relative NUE-Index, combined with leaf ureide and amino acid profiles, offers an integrated view of nitrogen uptake and mobilization, distinguishing genotypes that rely more on biological nitrogen fixation. Since the index considers both plant nitrogen accumulation and seasonal declines in soil mineral nitrogen, higher scores indicate stronger nodulation and fixation or better access to mineralizable nitrogen in low-input systems (Seo and Kim 2020). Since nodulation and biological nitrogen fixation were not directly assessed in this study, the relative NUE-Index should be viewed as an overall performance indicator combining nitrogen acquisition and utilization under the tested conditions, rather than a direct measure of nodulation traits. The strong associations with allantoin, a principal ureide transported from nodules, highlight its importance as a marker, since its levels reflect nitrogen transport and redistribution in legumes (Smith and Atkins 2002; Werner et al. 2010). Genetic signals near allantoin, asparagine, and arginine genes map to regions involved in transport, vesicle trafficking, and chloroplast function, compatible with known ureide loading and remobilization mechanisms (Rentsch et al. 2007; Tegeder and Rentsch 2010). The relationships involving the relative NUE-Index are considered in terms of carbon–nitrogen signaling, allocation, and physiological coordination that broadly affect nitrogen uptake and efficiency. However, future research explicitly measuring nodulation, N fixation, and root traits will be needed to fully understand the underlying mechanisms of these signals.

Notably, the lead relative NUE-Index loci (eg S08_49638740; *P* = 1.47 × 10^−7^; FDR = 0.00727 and S02_36911867; *P* = 5.80 × 10^−7^; FDR = 0.0109) lie near genes annotated as a protein phosphatase 2C and a trehalose-6-phosphate synthase/phosphatase, consistent with regulatory roles in carbon–nitrogen signaling and stress–responsive resource allocation. These findings suggest that tepary bean nitrogen metabolism involves specific modules for ureide transport and amino acid redistribution that vary among genotypes and can be tracked using metabolite data. The loci on chromosomes 2 and 8 associated with the index—linked to T6P and PP2C genes—support a carbon-regulated nitrogen-use mechanism. T6P acts as a sucrose-dependent signal coordinating growth and sink activity (O’Hara et al. 2013; Paul et al. 2018), while PP2Cs integrate ABA and stress pathways with carbon–nitrogen partitioning. These associations imply that nitrogen-use efficiency in tepary beans may depend more on carbon status, metabolic coordination, and stress signaling than traditional nitrate assimilation pathways—an adaptation for arid environments where photosynthate supply limits growth. While we annotated candidate genes within LD-supported intervals, conducting additional systems-level analyses could enhance the functional interpretation. Specifically, gene ontology enrichment and metabolomic network reconstruction would provide complementary insights into the biological processes and network connectivity associated with the mapped loci. We recommend these analyses as future steps, alongside multienvironment and multiyear validation, to assess locus stability and connect genetic associations with pathway-level structures. Overall, combining the index, leaf metabolite data, and candidate gene analysis offers a practical framework for selecting tepary genotypes with enhanced nitrogen fixation and efficient nitrogen remobilization.

### Flowering-time genetics as a lever to optimize nitrogen capture and residue management

Flowering time is vital for deploying cover crops because early or late flowering can change biomass duration, ground cover, and how well they fit into crop rotations. Flowering time is widely considered a complex trait that can involve many loci and environment-dependent effects. In this dataset, however, DTF variation includes a major-effect component, represented by the lead association at S03_40020268 (*P* = 2.96 × 10^−10^; FDR = 1.46 × 10^−5^), which should be interpreted as evidence for a hierarchical architecture (a major locus embedded within a broader polygenic background) rather than as evidence of single-locus control across environments. The high minor allele frequency of the lead locus supports its biological relevance and reduces the likelihood that the signal reflects a rare-allele artifact. The DTF-related gene on chromosome 3, Phacu.CVR.003G300600 encodes a BTB domain–containing protein (H-BTB1). The DTF locus does not overlap with the genome-wide significant biomass loci identified in this panel (Supplementary Table 4), suggesting no direct genetic link between the flowering-time association and biomass accumulation. As these candidate genes are inferred from physical proximity within an LD-supported interval, this BTB gene is presented as a testable hypothesis rather than a confirmed causal regulator; validation will require local LD/haplotype analysis and functional evidence. BTB proteins serve as substrate-recognition components of CUL3 E3 ubiquitin ligases, which control hormonal, environmental, and developmental processes (Ban and Estelle 2021; Chung et al. 2025). Recent work shows that BTB–CUL3 complexes can influence temperature-responsive flowering by facilitating the degradation of repressors such as SVP, thereby accelerating flowering under warmer conditions (Jin et al. 2024). Although our study does not directly test the underlying mechanism or genotype × environment responses, the proximity of the lead DTF association to a BTB-domain gene supports this locus as a plausible, testable candidate region for flowering-time regulation under the field conditions examined. This trait plasticity makes it more adaptable for flexible planting schedules, ensuring reliable biomass production and preventing early flowering. The limited overlap between DTF and biomass/NUE genes suggests minimal trade-offs when combining traits. The lead flowering-time association on chromosome 3 (S03_40020268) suggests that a locus with a large effect significantly influences DTF variation in this panel under the tested conditions. Nonetheless, this finding does not exclude the presence of other smaller-effect loci, loci below the detection threshold, or loci that may be expressed in different environments. Therefore, flowering time should be considered a trait primarily governed by a major-effect locus within a broader polygenic framework background. Future multienvironment trials and local haplotype-based analyses around the Chr03 region will aid in quantifying environmental sensitivity and determining if other loci also influence flowering time in this panel. For cover cropping, delaying flowering extends the vegetative phase, allowing more biomass and nitrogen capture before termination. Conversely, earlier flowering or a faster reproductive transition can be useful for rotation cycles that require shorter field time. Since legume residues usually have low C: N ratios, timing termination at late vegetative or early reproductive stages can balance the need for enough structural material to protect soil with residues that mineralize at rates matching the nitrogen needs of the next crop (Koudahe et al. 2022). The benefit of tepary bean is that the BTB-related locus offers a manageable way to tweak flowering time, which can be combined with biomass and nitrogen efficiency traits without clear trade-offs. This allows designing ideotypes suited to local summer windows and management goals, maintaining residue quality and nitrogen provision (Moghaddam et al. 2021; Bornowski et al. 2023; Ravelombola et al. 2024).

From an agronomic perspective, flowering time can influence the duration of vegetative growth and thereby affect biomass accumulation in cover-crop settings. However, our current GWAS results do not demonstrate a shared genetic basis between the lead DTF locus and the biomass loci. Therefore, any phenology–biomass relationship should be considered a physiological hypothesis that must be evaluated directly in multienvironment trials and/or through joint genetic models explicitly testing pleiotropy or causal mediation. Nonetheless, biomass–reproduction trade-offs also manifest in plant architecture and developmental timing, including canopy traits associated with soil water conservation, reproductive-phase processes, such as nutrient allocation, pod-filling duration, and sink development phenology. These traits should be specifically studied in future multienvironment experiments. Under drought and heat-stress conditions, breeding priorities often shift from simply maximizing biomass to optimizing biomass allocation and maintenance across developmental stages. A practical approach combining marker-assisted selection targeting phenology loci (DTF locus) with genomic selection to capture the small-effect, polygenic contributions to traits such as biomass, amino acid content, and nitrogen use efficiency. Such multitrait genomic prediction models would help integrate correlated traits and address trade-offs (eg between biomass and flowering during water scarcity) across diverse environments, thereby facilitating the assessment of conditional neutrality and enhancing the exploitation of existing adaptive variation within tepary germplasm.

## Conclusion

This study presents a comprehensive genomic analysis of biomass yield, phenology, nitrogen-use–related physiology, and leaf amino acid metabolism in *P. acutifolius*, providing insights relevant to its development as a resilient summer cover crop. Biomass-associated loci linked to structural cell wall and developmental functions provide candidate regions for improving vegetative growth in hot, dry environments. The relative NUE-Index, considered here as an integrated performance metric of nitrogen acquisition and utilization under the tested conditions, identified genomic regions near trehalose-6-phosphate and phosphatase signaling genes, supporting a role for coordinated carbon–nitrogen regulation in NUE-related trait variation. Additionally, a major flowering-time locus on chromosome 3 offers a tractable opportunity to adjust phenology to optimize biomass duration and nitrogen capture or to synchronize termination with cropping constraints. Leaf ureide and amino acid profiles, including substantial variation in allantoin, provide complementary physiological context and testable hypotheses for nitrogen status and nitrogen turnover. Overall, these trait-associated loci in P. acutifolius provide a genomic foundation for marker-assisted selection, ideotype development, and introgression into interspecific breeding programs to improve nitrogen management, soil health, and resilience in sustainable farming systems.

## Supplementary Material

jkag119_Supplementary_Data

## Data Availability

All sequencing data for tepary bean accessions have been submitted to the National Center for Biotechnology Information (NCBI) under the BioProject accession PRJNA1416895 (Alla and Joshi 2026a). Filtered GBS SNP data are available on Figshare: https://doi.org/10.6084/m9.figshare.31158676 (Alla and Joshi 2026b). Supplemental material available at G3 online.
