# Supplementary material for: Genotype-by-sequencing–enabled genome-wide association studies reveal genetic architecture of biomass and nitrogen modulation in tepary bean (Phaseolus acutifolius)
Source: G3 (Bethesda). 2026 May 5;16(7):jkag119. doi: 10.1093/g3journal/jkag119 (PMC13334183; doi:10.1093/g3journal/jkag119)
Supplement: jkag119_Supplementary_Data [file jkag119_supplementary_data.zip › Supplemental_Material_Legends_G3-2026-406764.docx]

**Supplemental material - legends**

**Supplementary Figure 1**. Genome‑wide LD decay (r² vs. physical distance) estimated from the GWAS SNP dataset for the diversity panel. The fitted decay curve indicates LD decays to r² = 0.2 at ~14 kb, supporting the use of a ±10 kb candidate‑gene window.

**Supplementary Fig. 2.** Frequency distributions (histograms) of leaf free amino-acid traits across accessions. Each panel shows among-accession variation for a single amino acid; check genotypes are indicated by vertical reference lines where applicable.

**Supplementary Figure 3. GWAS for GABA, glutamic acid, and glycine.**

Manhattan plots for (A) GABA, (B) glutamic acid, and (C) glycine. The genomic coordinates (x-axis) and –log₁₀(p-values) (y-axis) are shown. Significant SNPs exceeded the –log₁₀p = 6 threshold and were used for candidate gene identification.

**Supplementary Figure 4. GWAS for isoleucine, leucine, and lysine.**

Manhattan plots for (A) isoleucine, (B) leucine, and (C) lysine, with significant SNPs marked above the genome-wide threshold. These associations identify trait-specific genomic regions underlying branched-chain amino acid metabolism.

**Supplementary Figure 5. GWAS for methionine, phenylalanine, proline, serine, and tyrosine.**

Manhattan plots for (A) methionine, (B) phenylalanine, (C) proline, (D) serine, and (E) tyrosine. Significant SNPs (–log₁₀p ≥ 6) were used to identify candidate genes related to amino acid biosynthesis and turnover.

**Supplementary Table 1. Tepary bean accessions and commercial checks used in this study.** Includes USDA‑NPGS accession numbers, origin, and market‑type descriptions for all evaluated materials.

**Supplementary Table 2. Adjusted phenotypic values (BLUPs) for biomass, Relative NUE‑Index, and days to flower.** Values generated from linear mixed models used as input for GWAS.

**Supplementary Table 3. Adjusted phenotypic values (BLUPs) for 20 leaf amino acids.** These adjusted means were used for all amino‑acid GWAS analyses.

**Supplementary Table S4**. **Lead GWAS loci (QTLs) for biomass, days to flowering, and the Relative NUE‑Index**. For each lead SNP, the table reports chromosome position, raw and FDR‑adjusted p‑values, effect/MAF, and the full set of annotated gene models within the LD‑supported ±10 kb window surrounding the lead SNP.

**Supplementary Table 5. Genome‑wide significant SNPs associated with leaf amino acids, including candidate genes and functional annotations.** Lists SNPs, chromosome locations, raw and FDR‑corrected p‑values, associated genes, and predicted gene functions.
