# Supplementary figures and images for: Genotype-by-sequencing–enabled genome-wide association studies reveal genetic architecture of biomass and nitrogen modulation in tepary bean (Phaseolus acutifolius)
Source: G3 (Bethesda). 2026 May 5;16(7):jkag119. doi: 10.1093/g3journal/jkag119 (PMC13334183; doi:10.1093/g3journal/jkag119)

Genome-wide LD decay

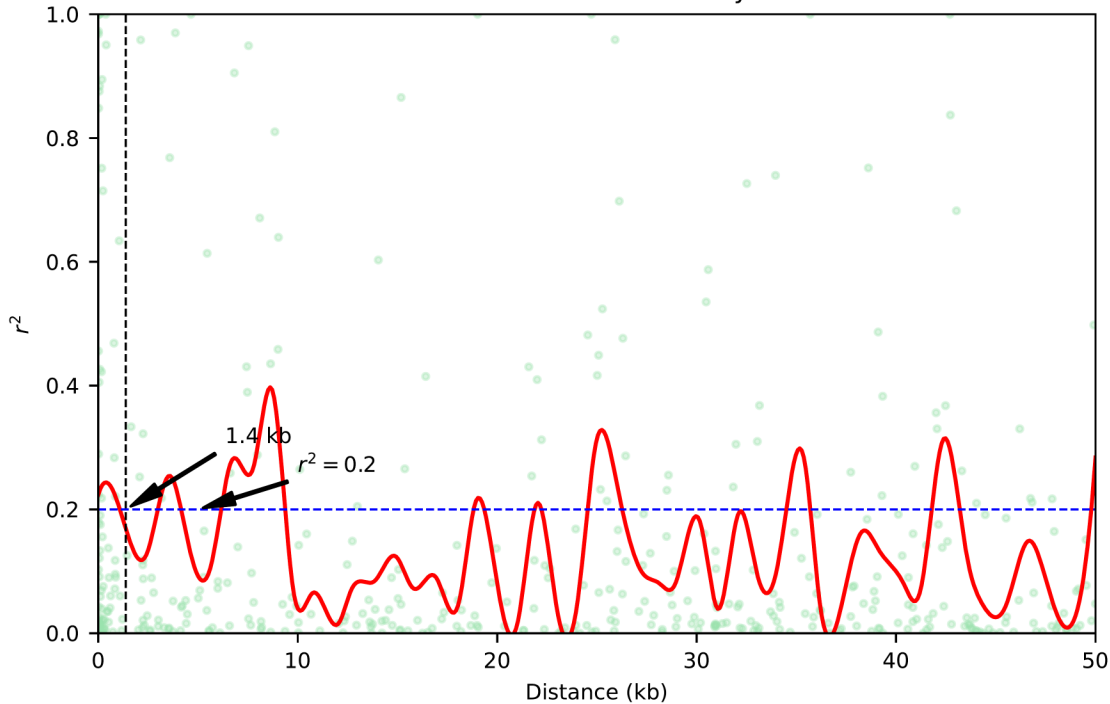

Supplement: jkag119_Supplementary_Data [file jkag119_supplementary_data.zip › Supplemental_Fig_1_G3-2026-406764.pdf]

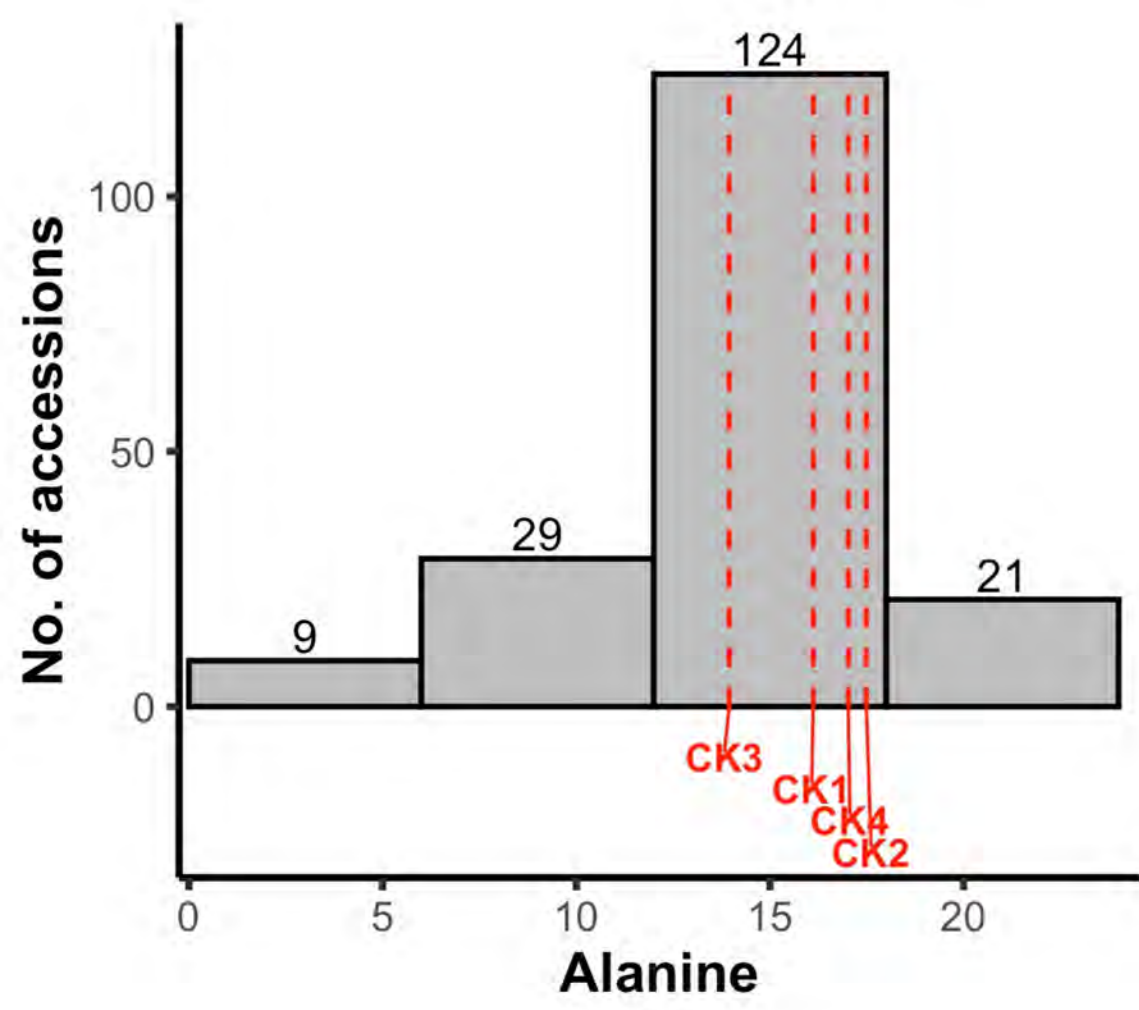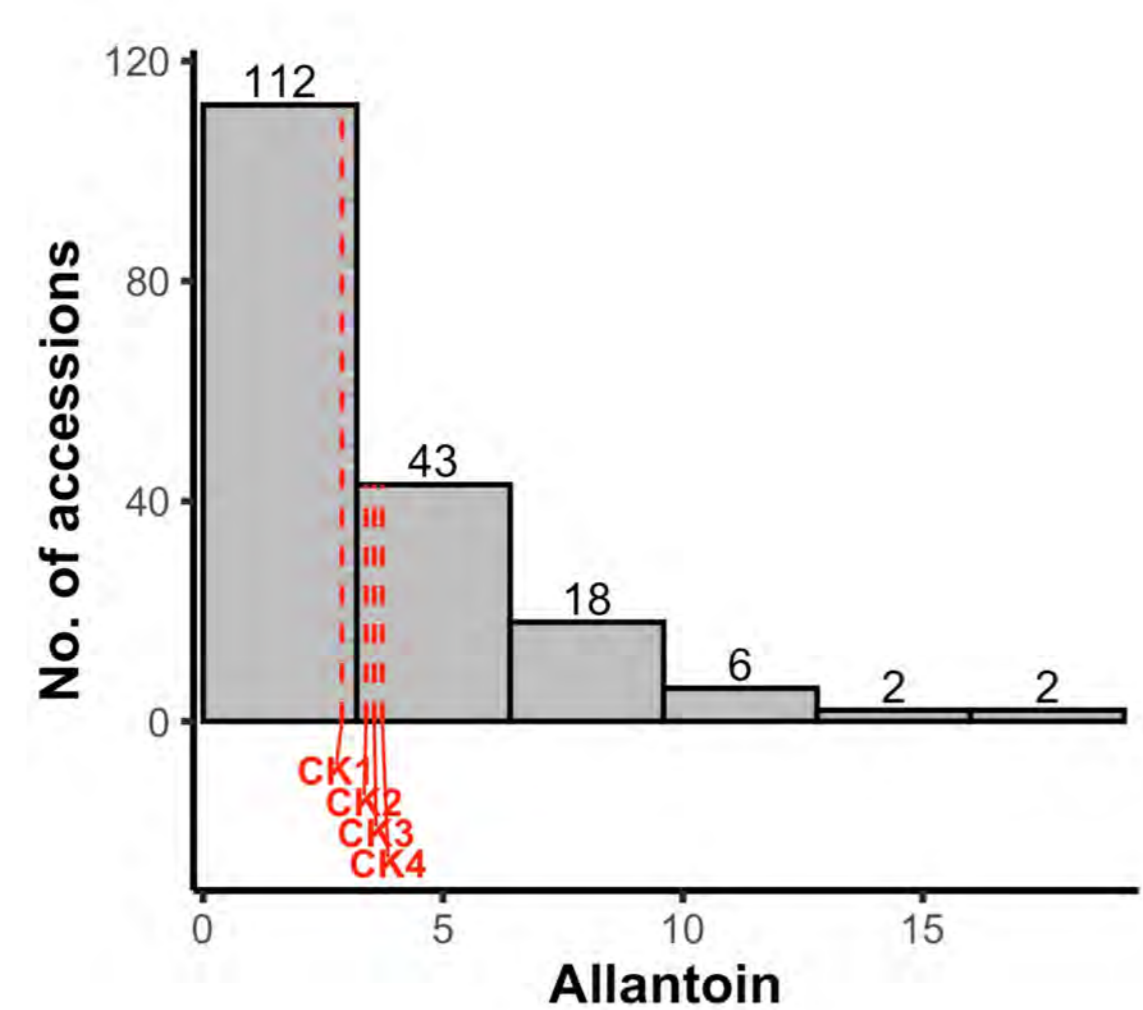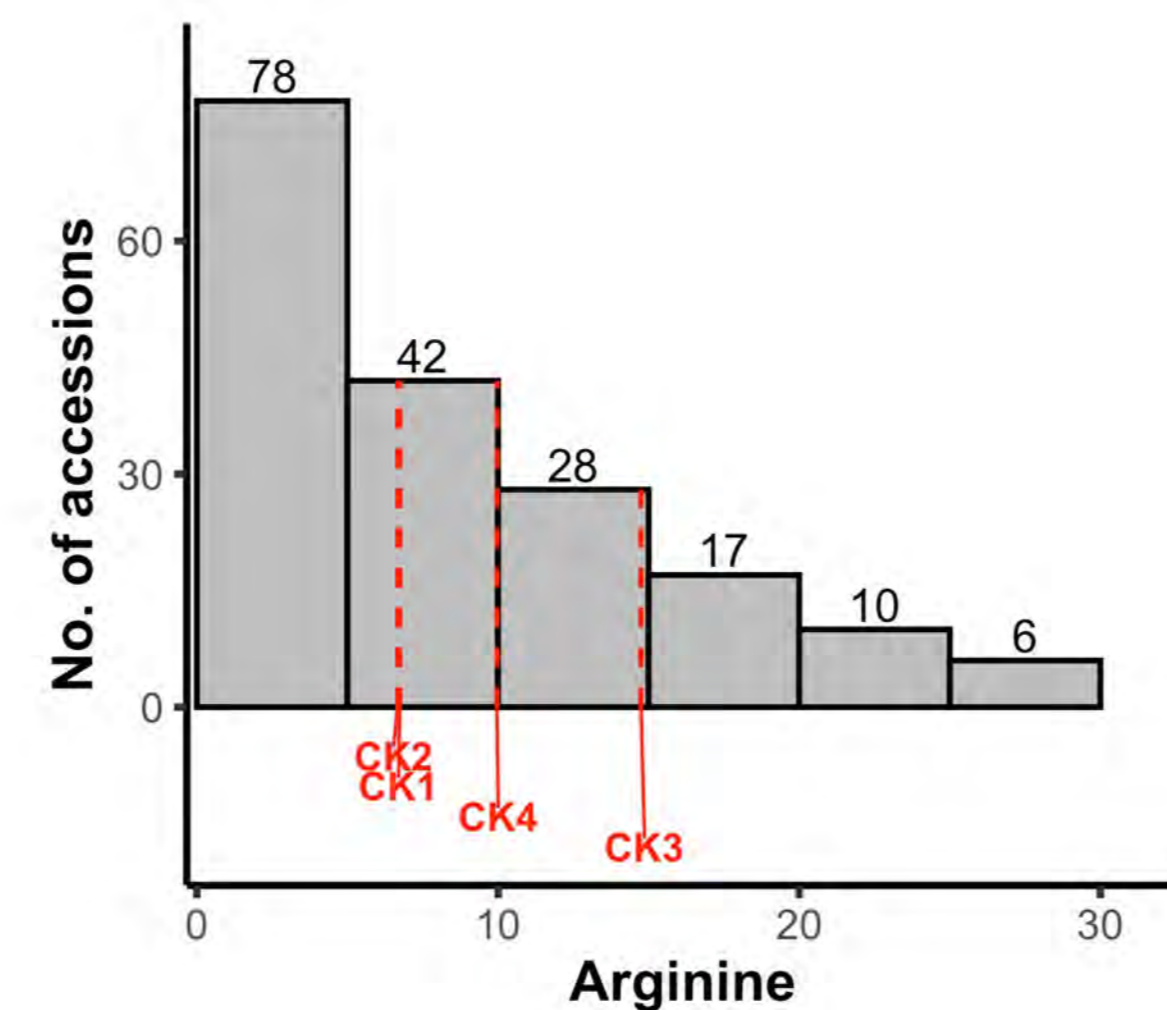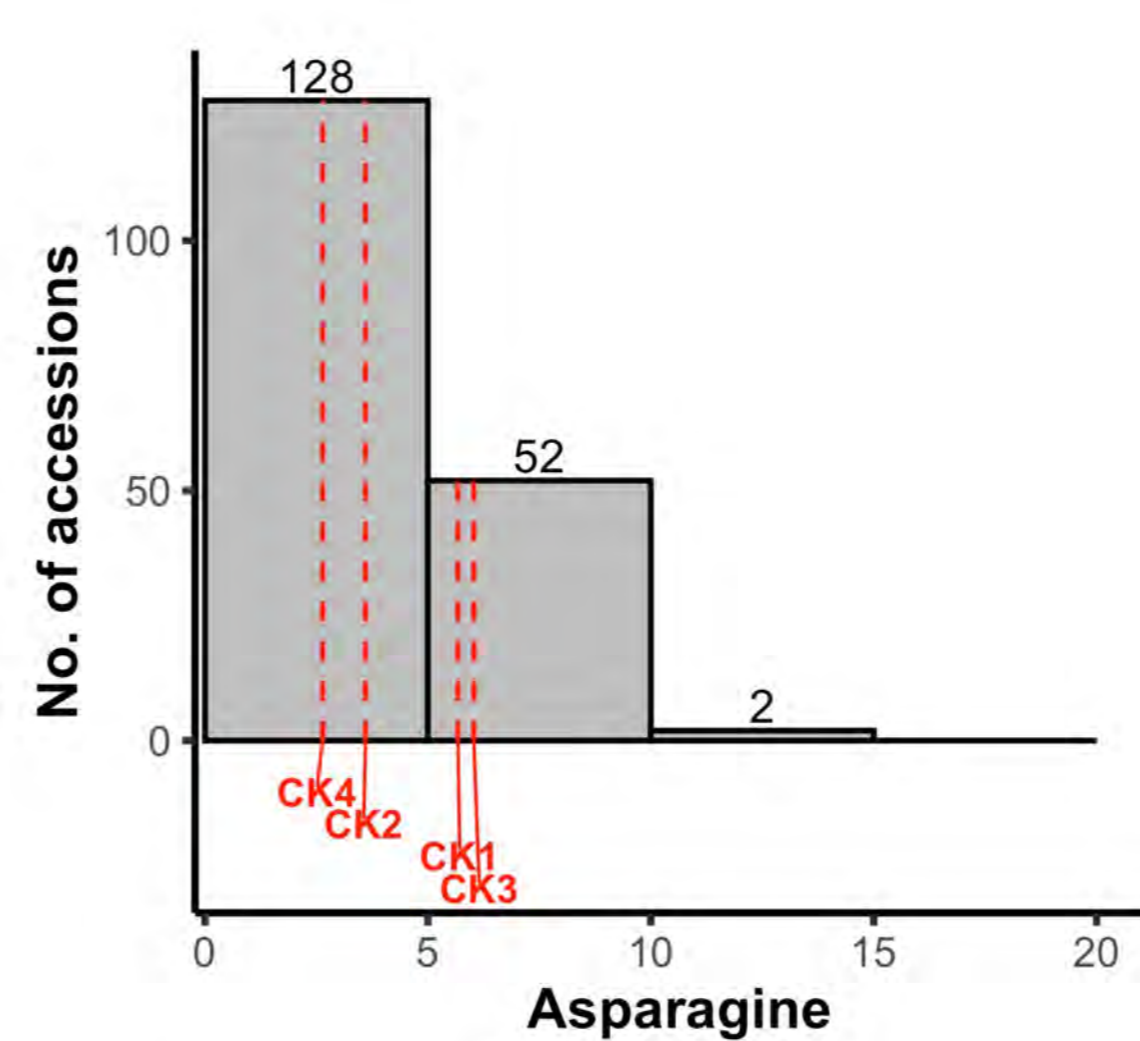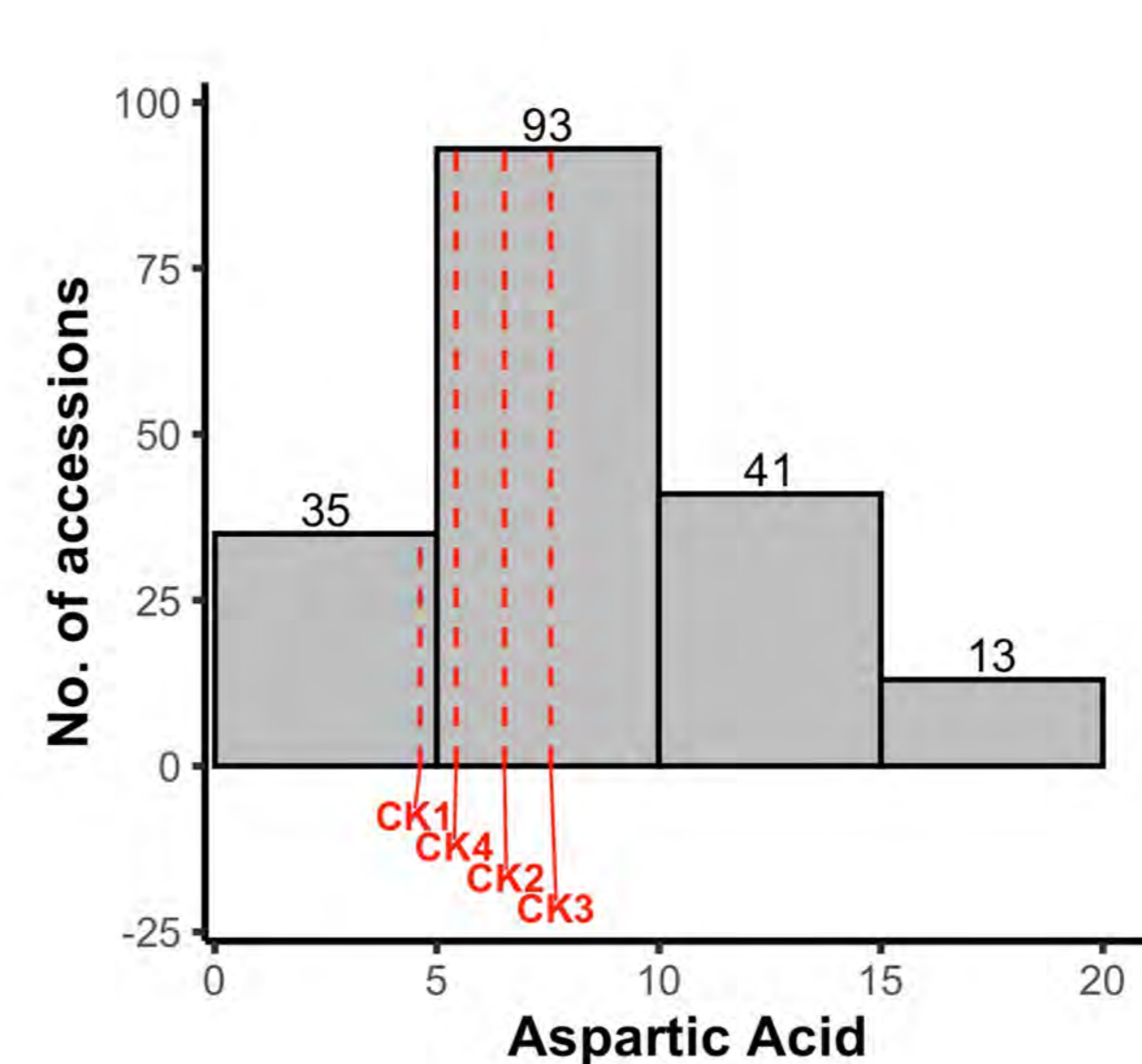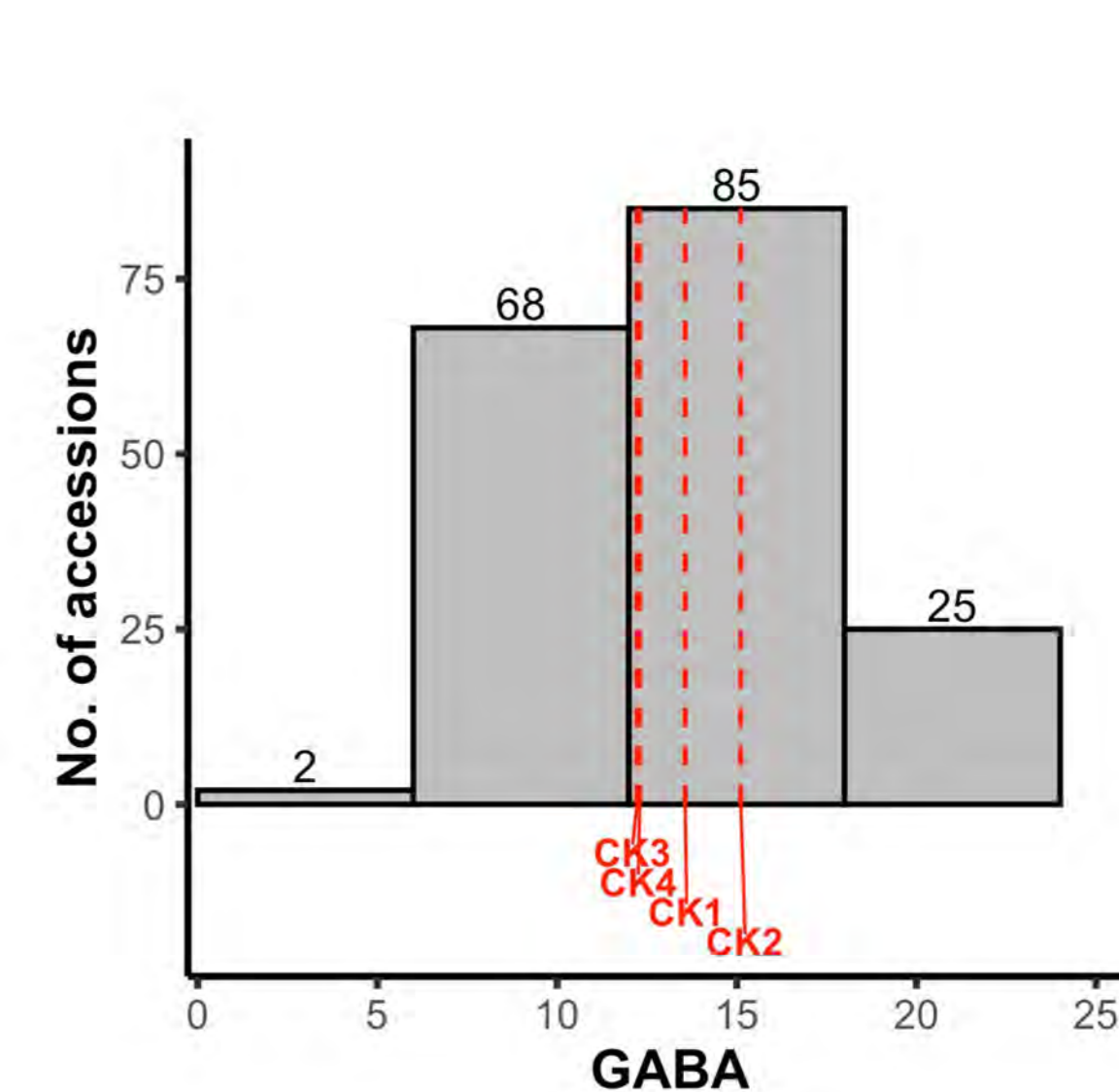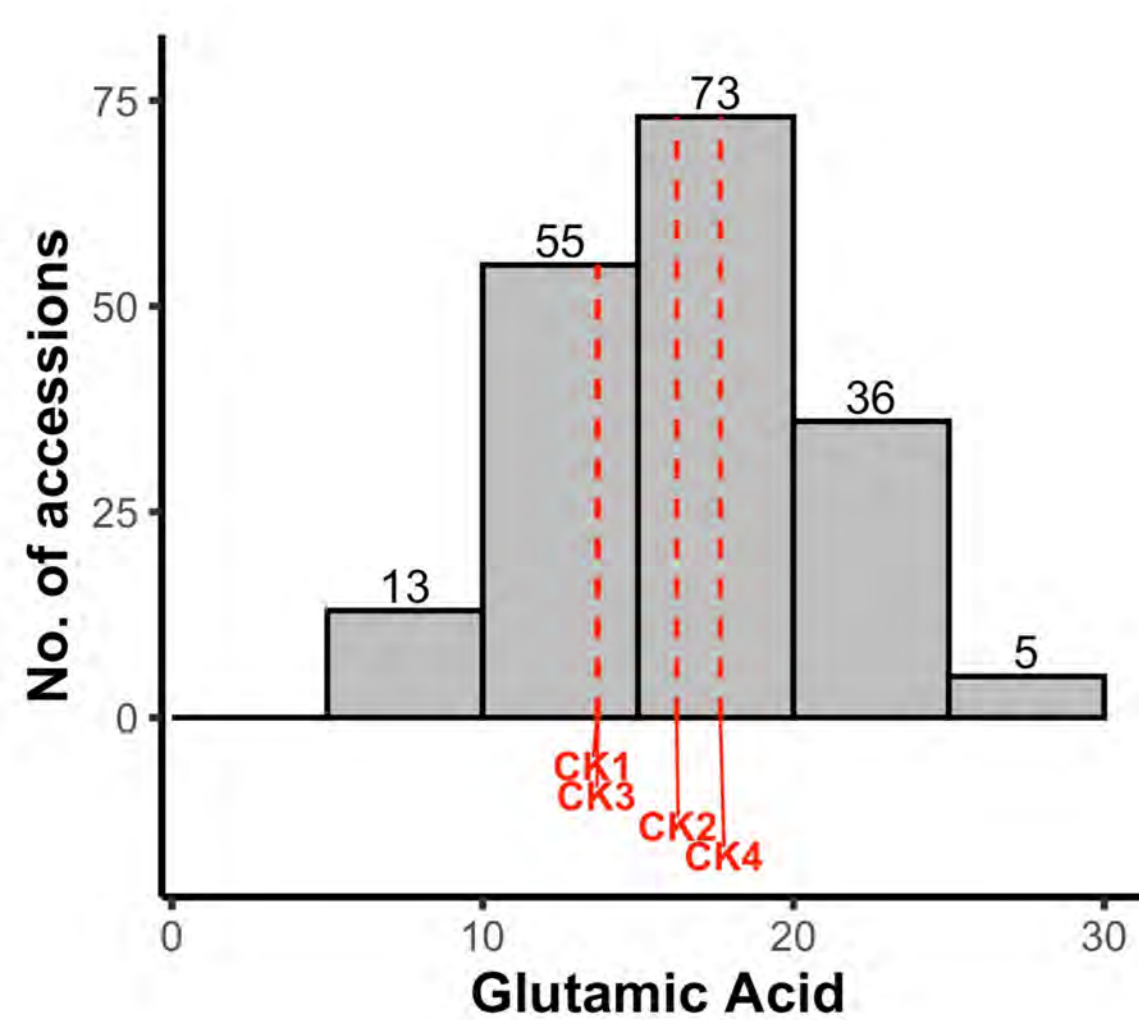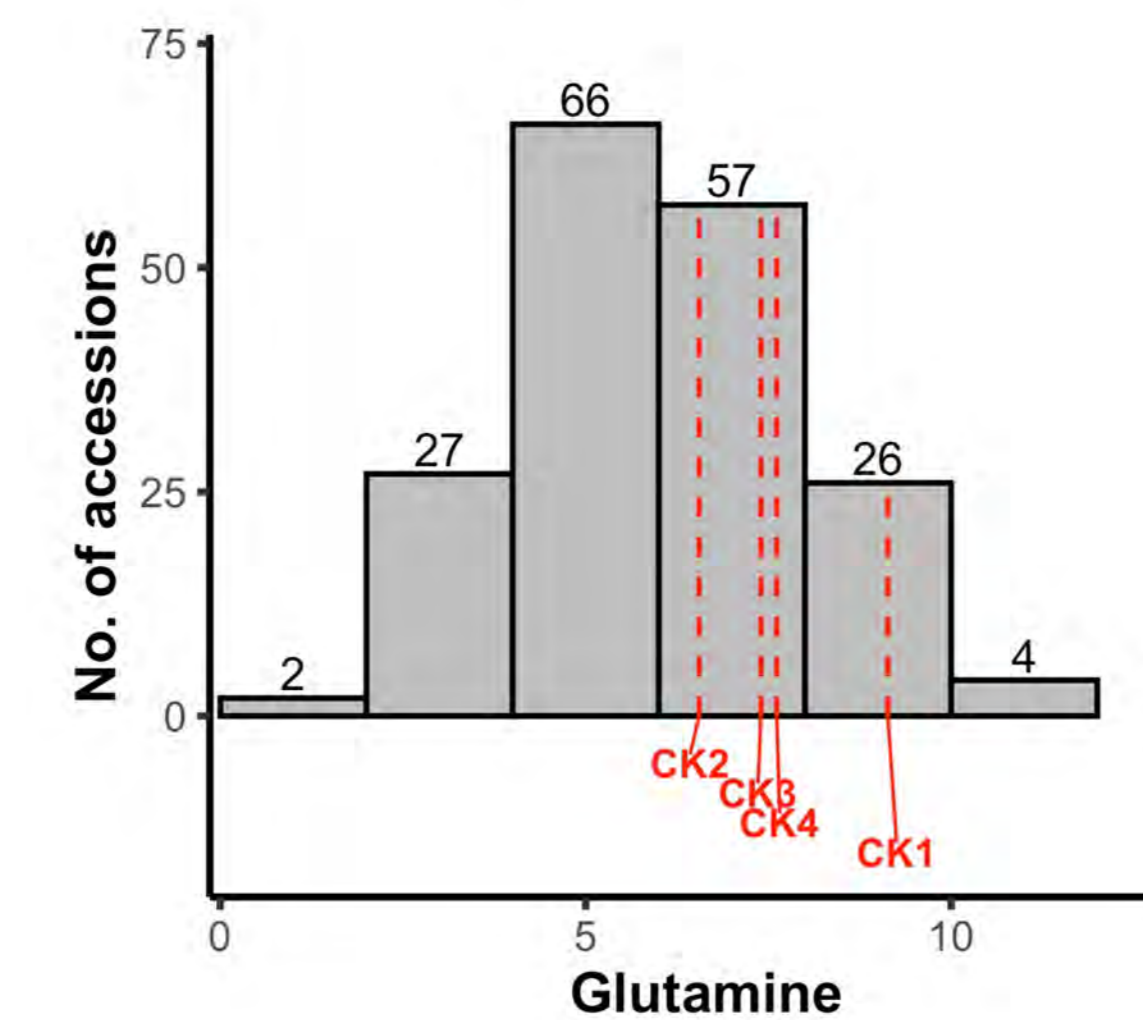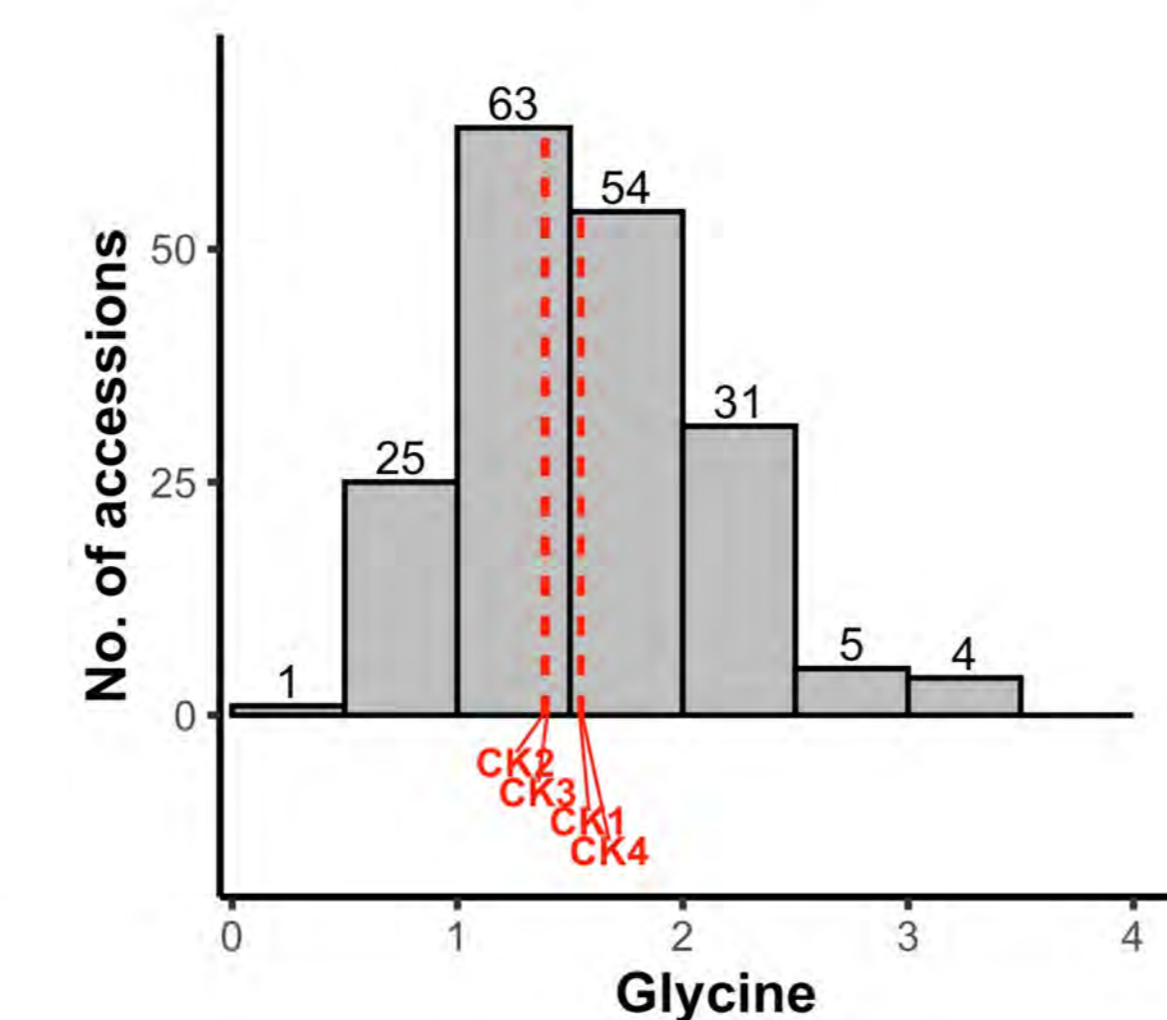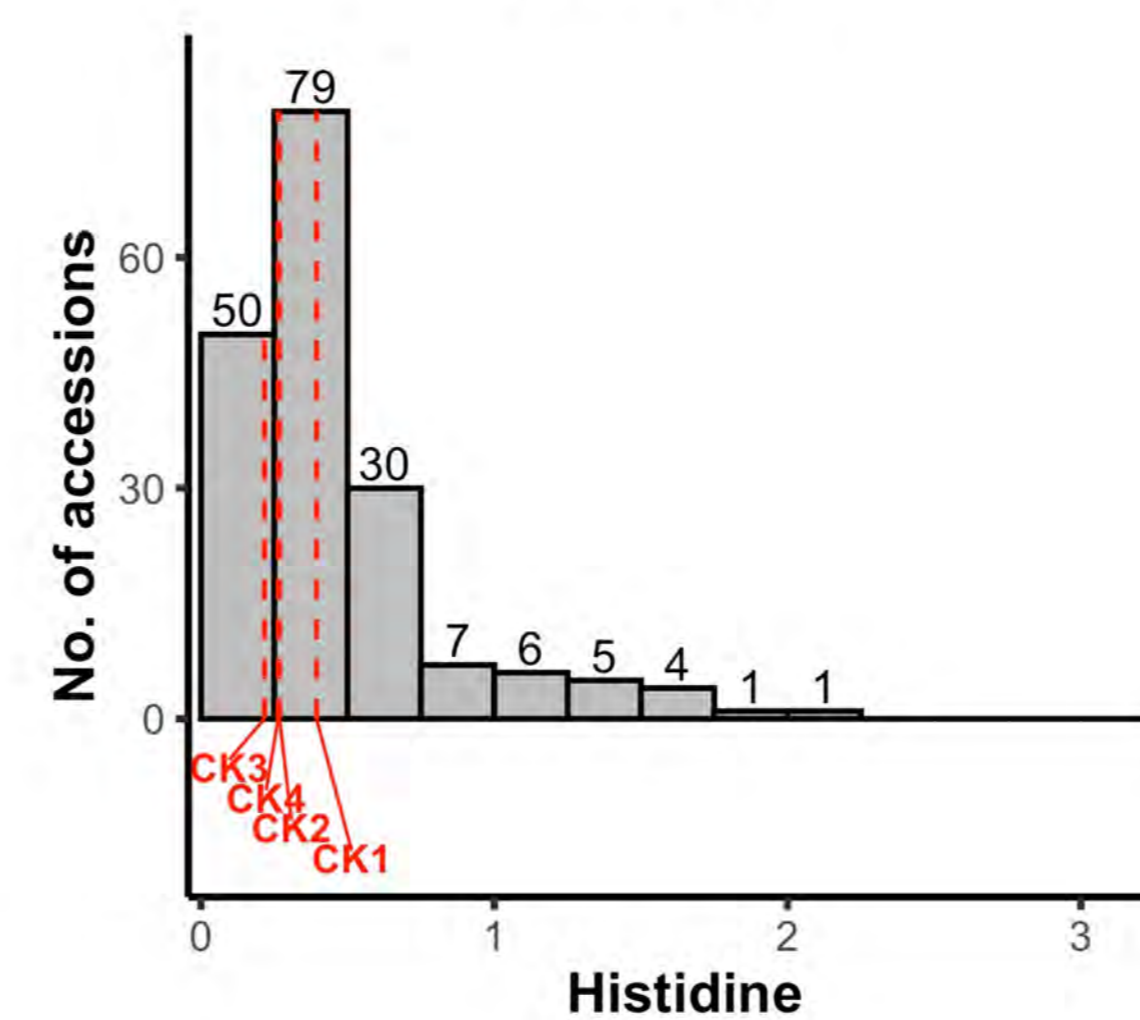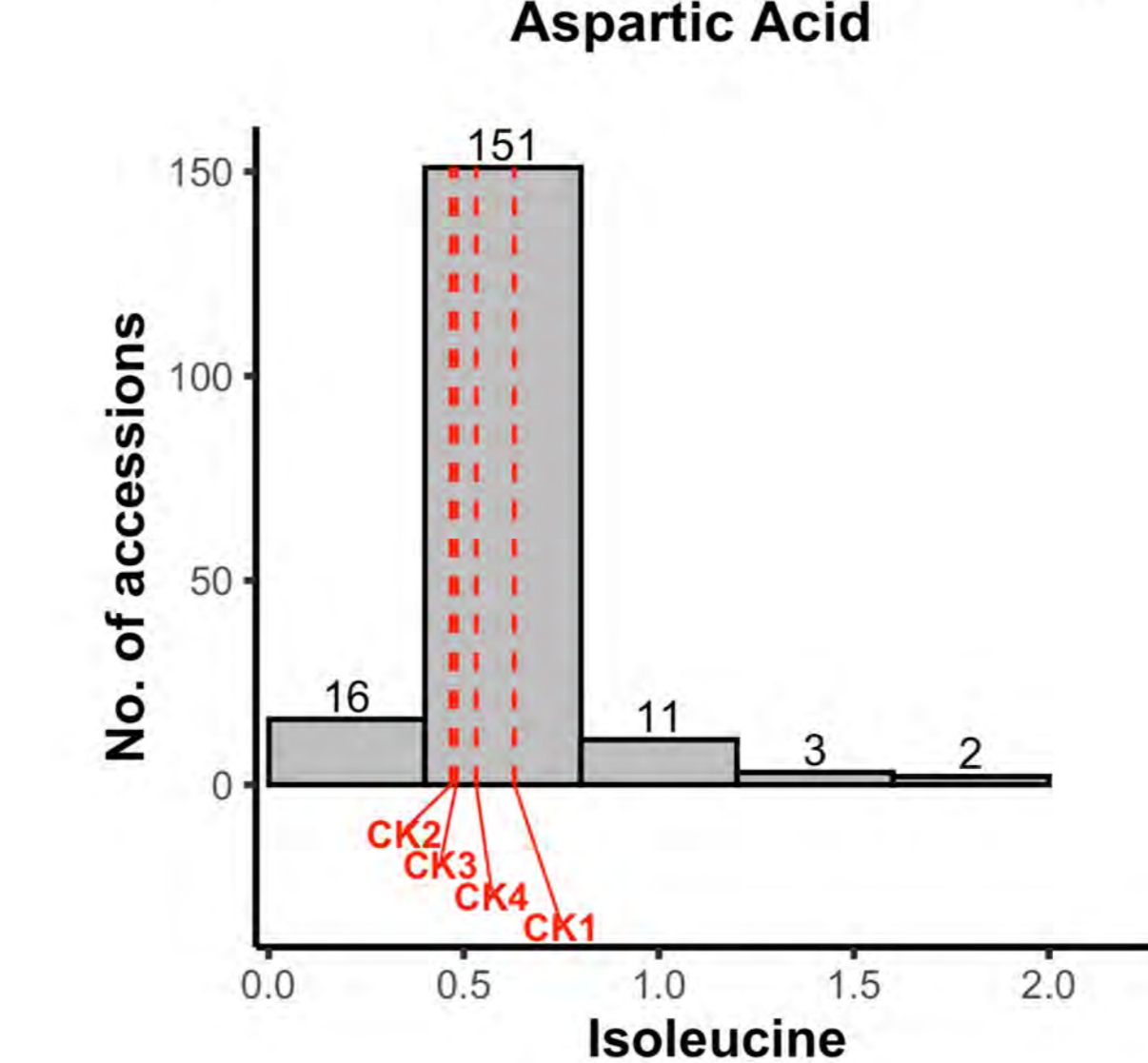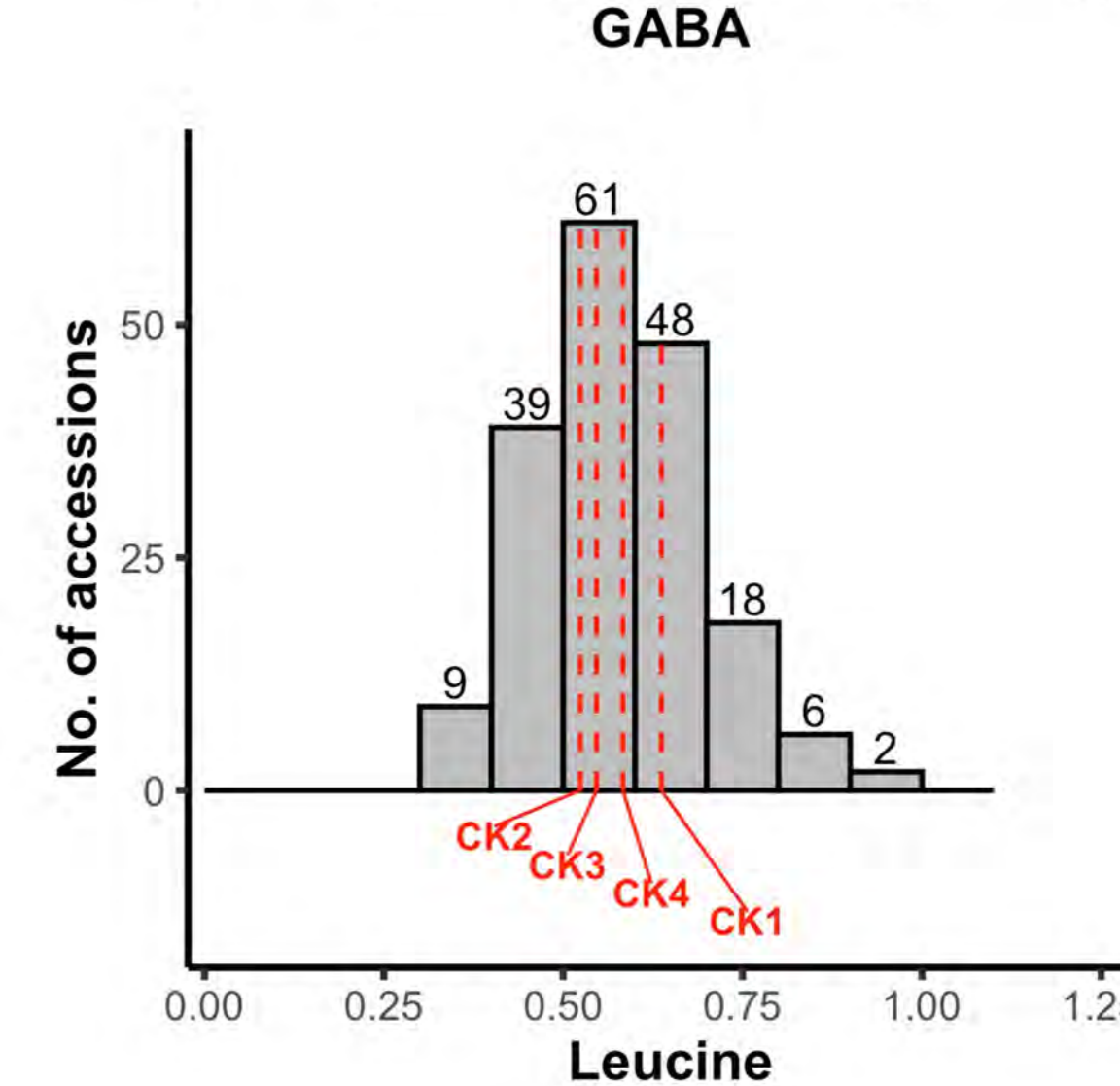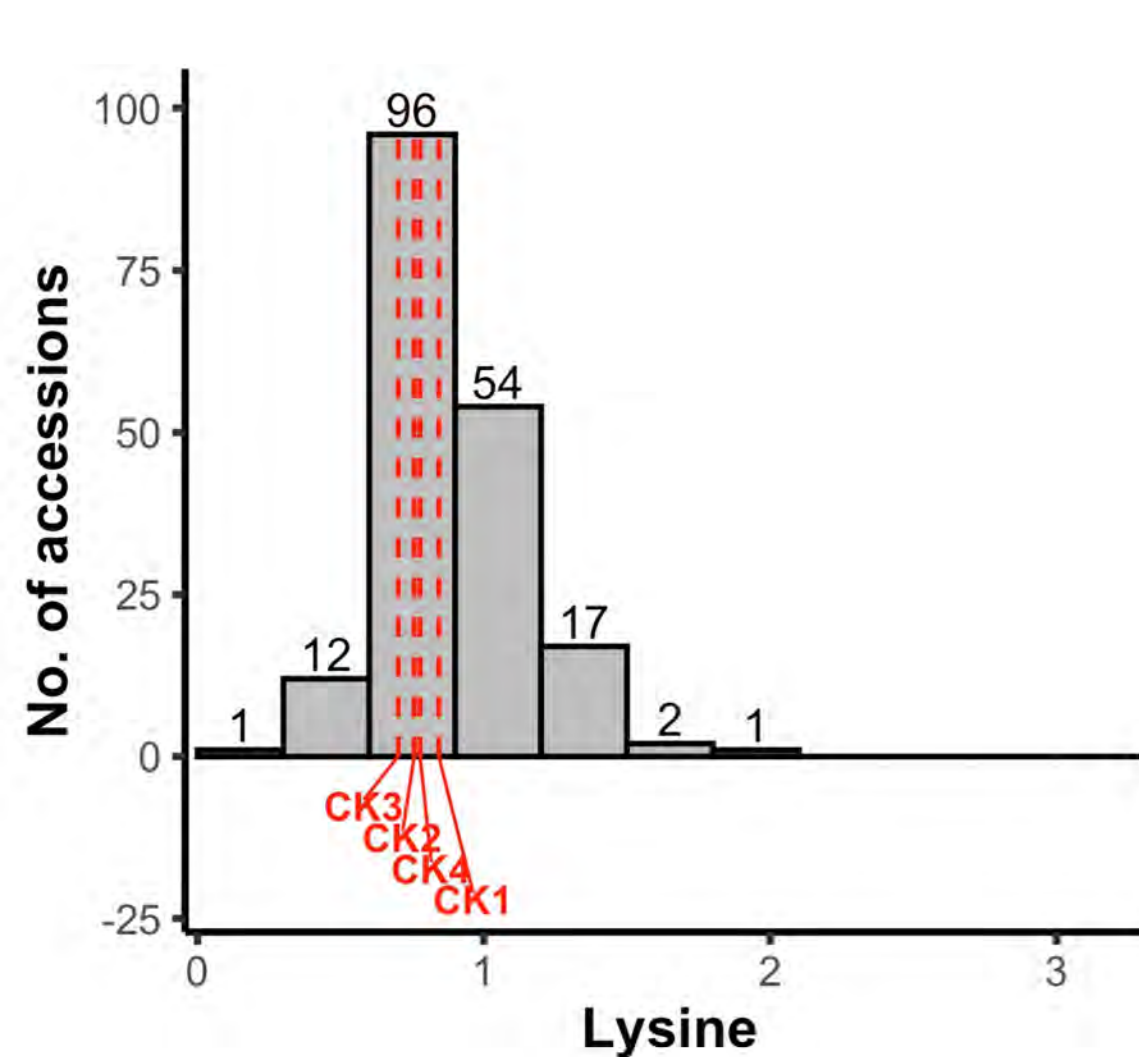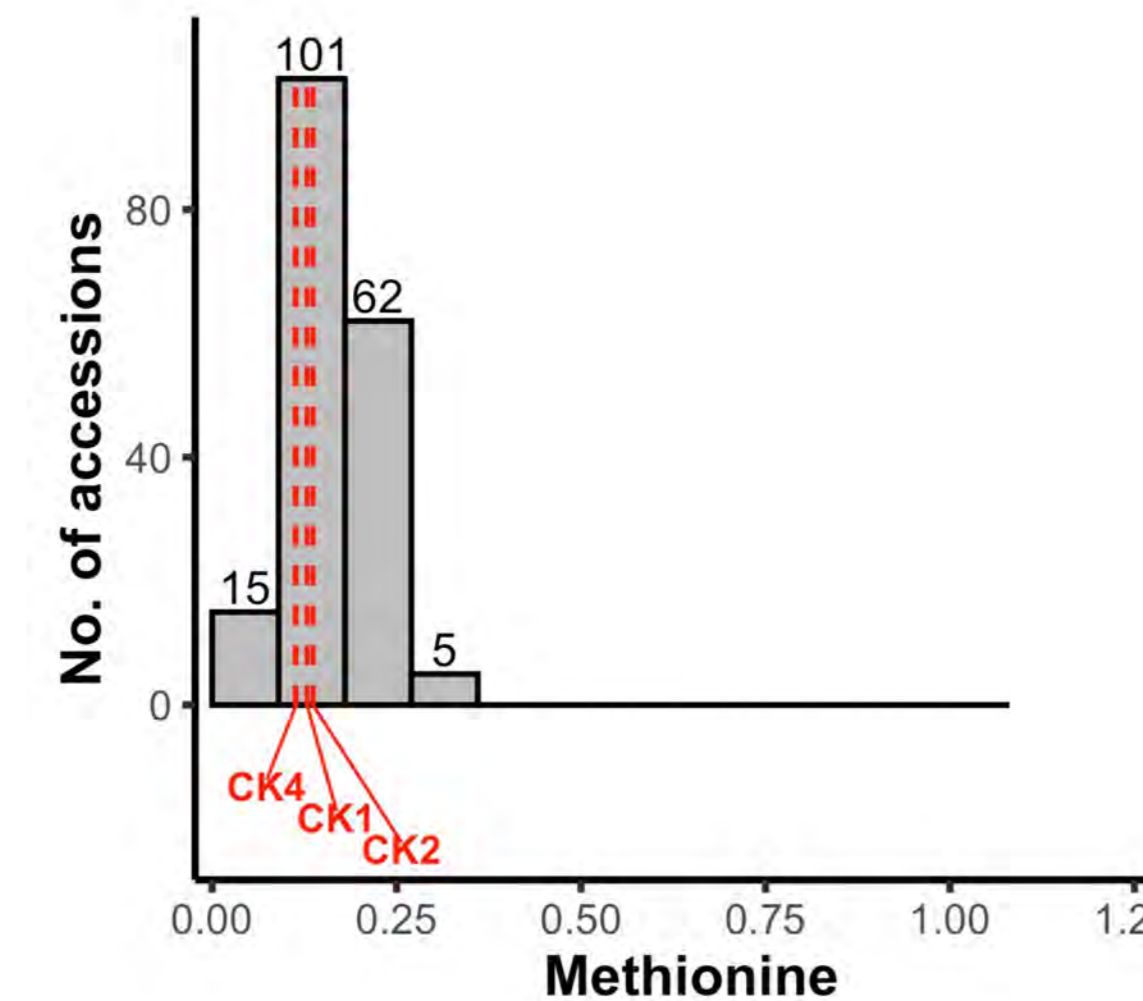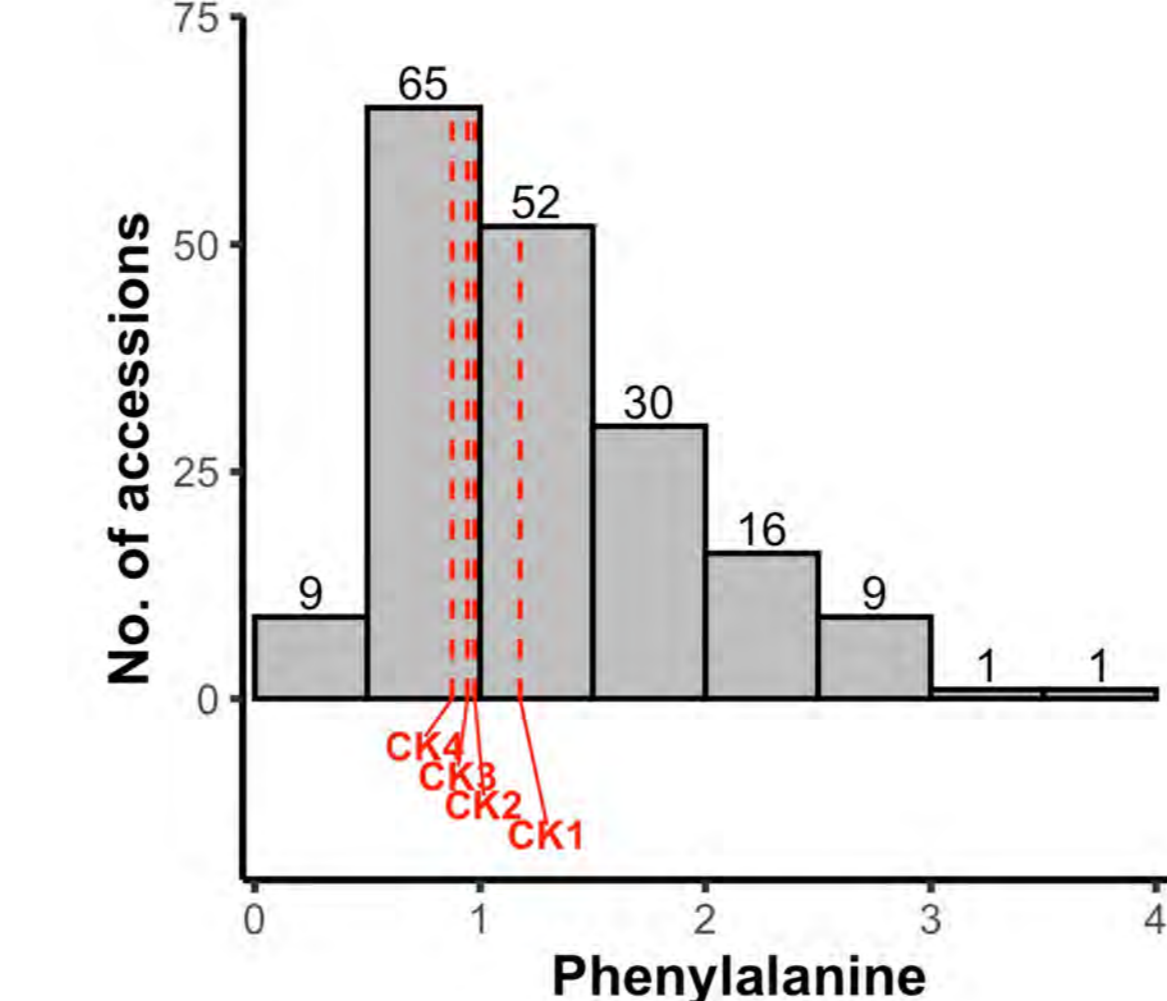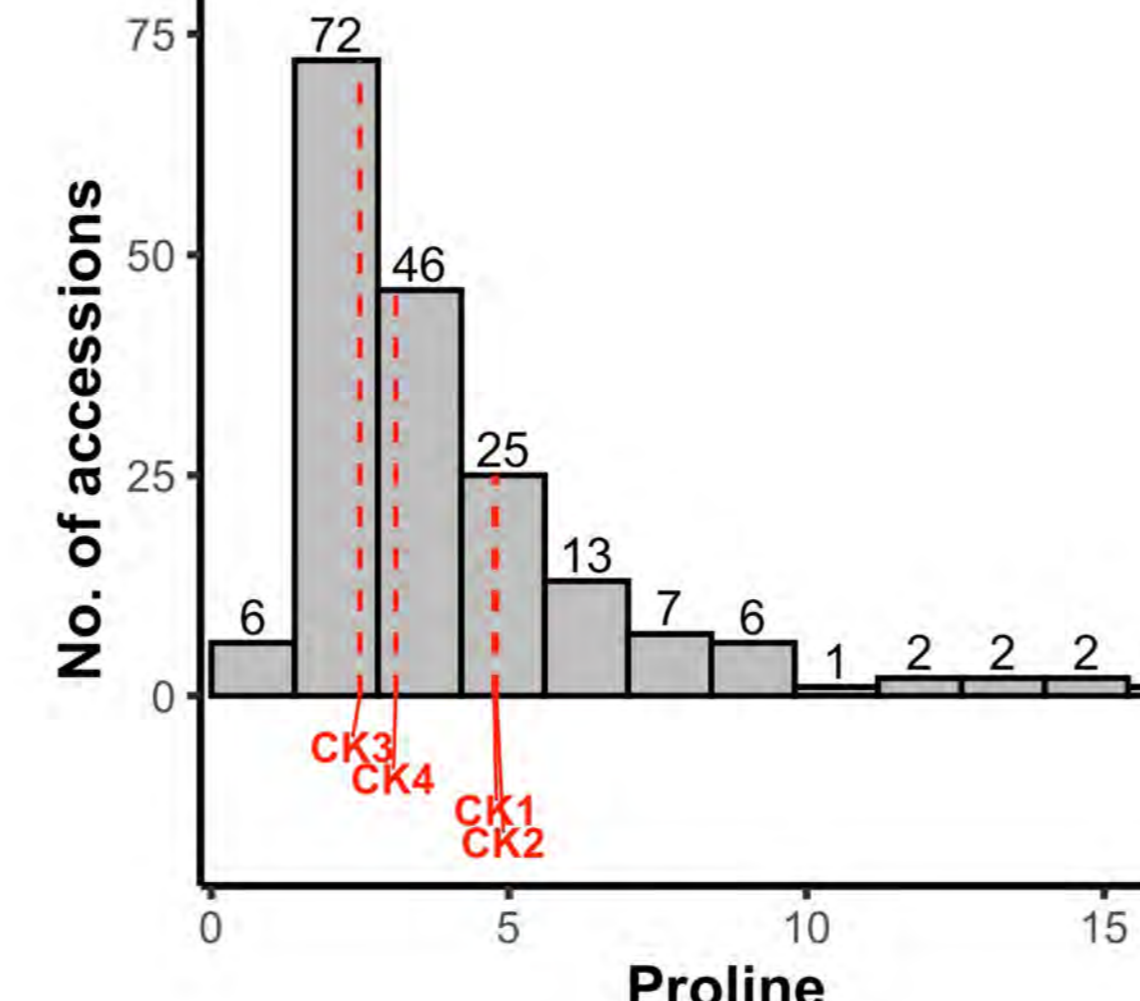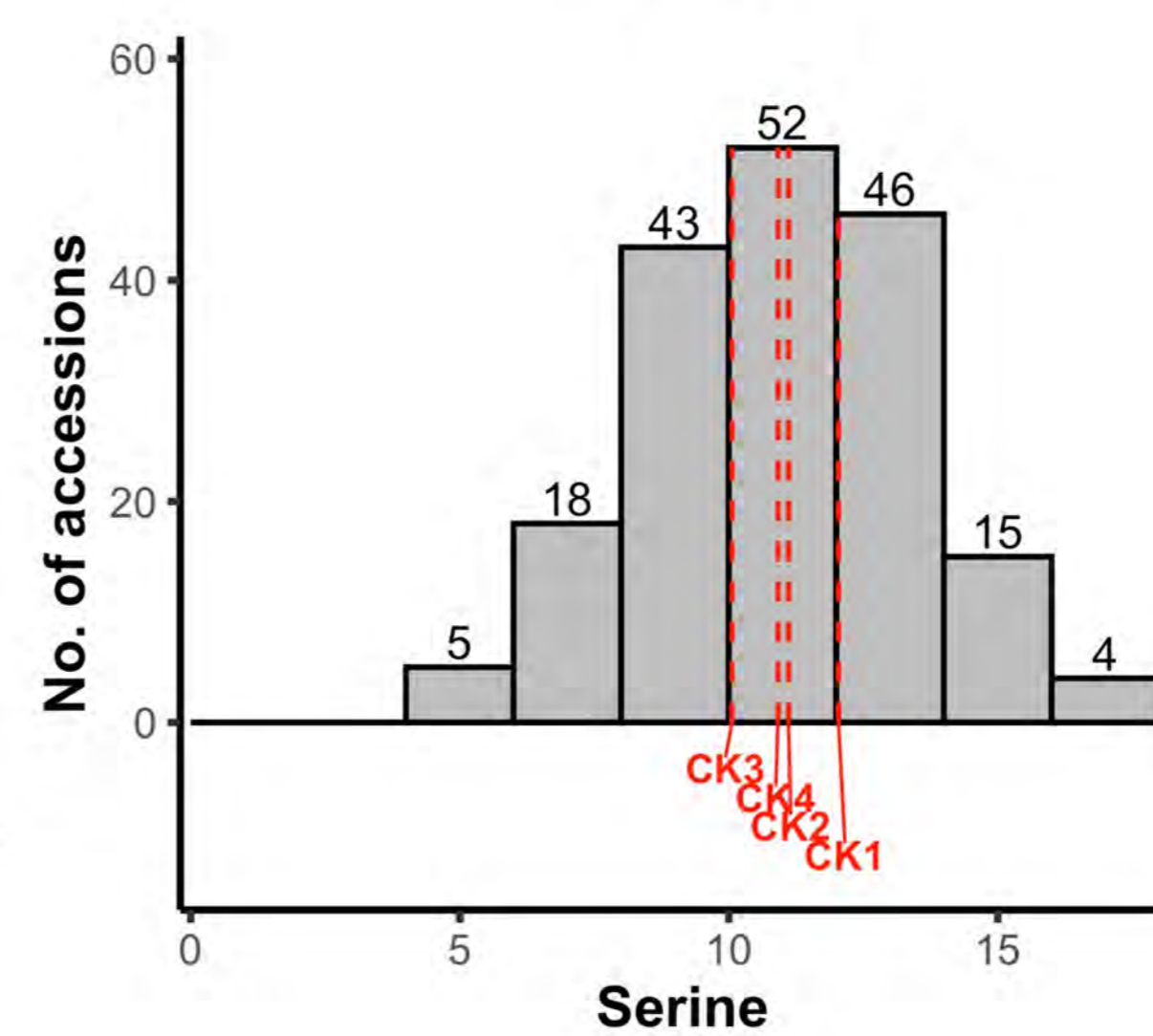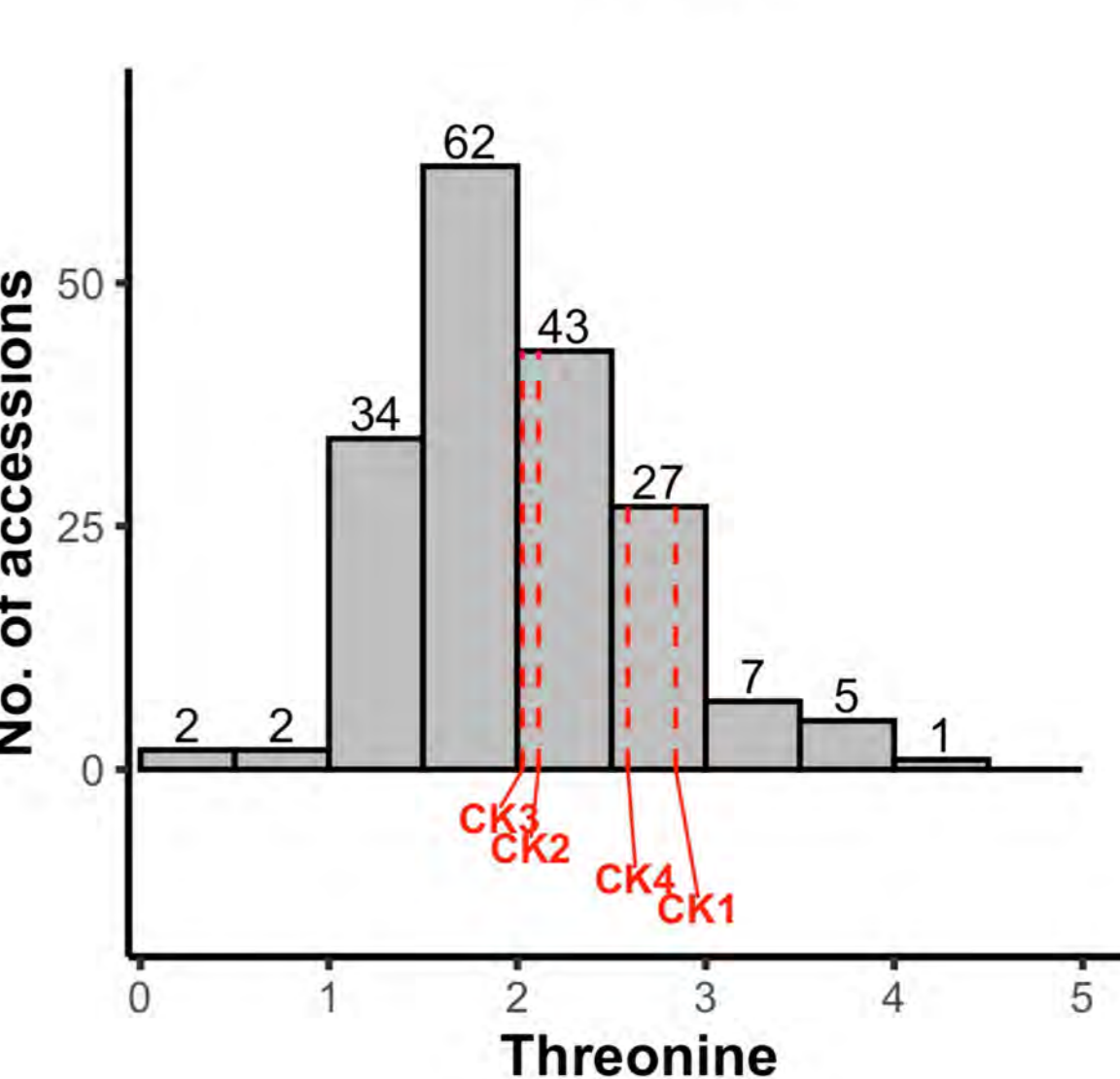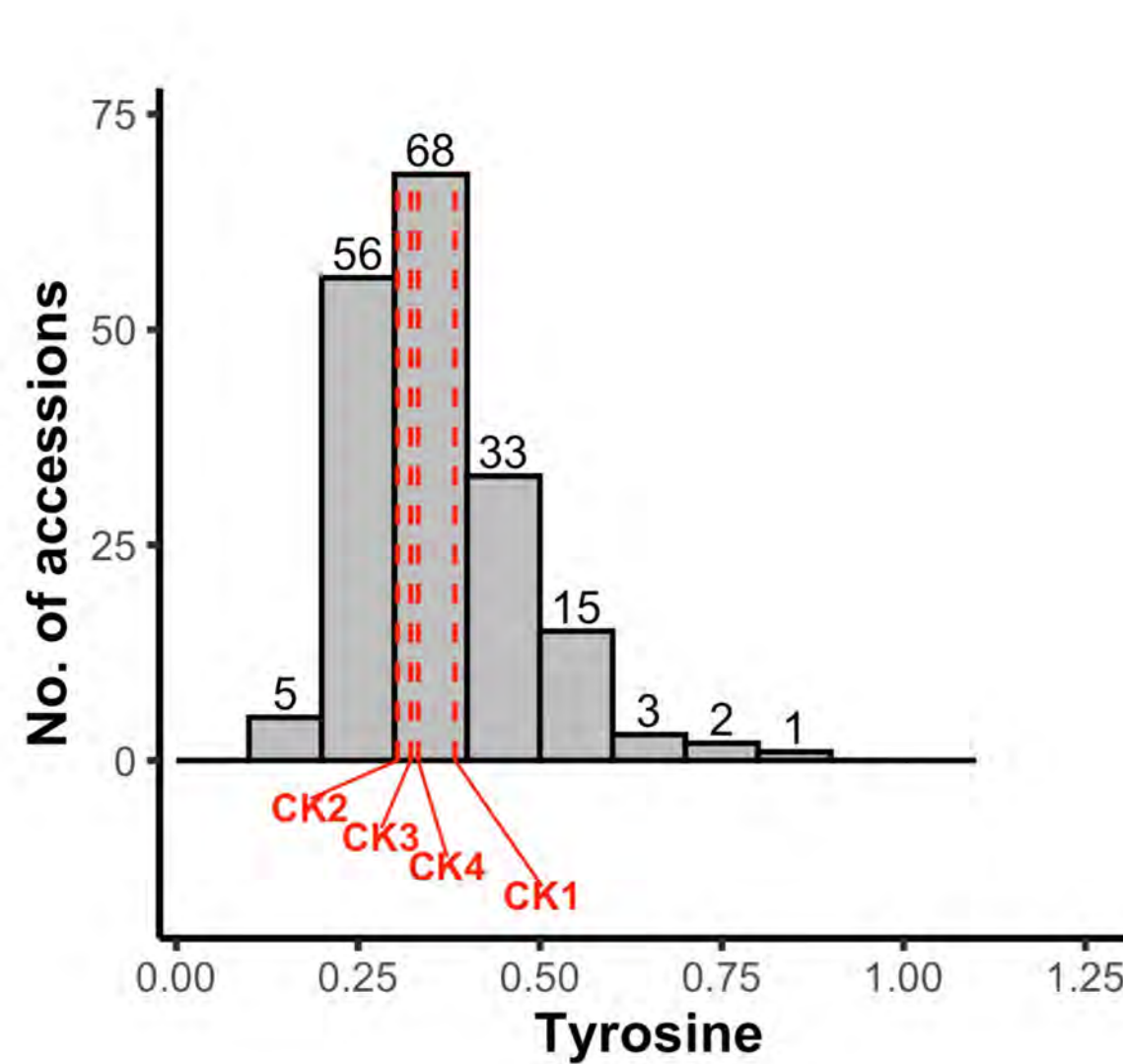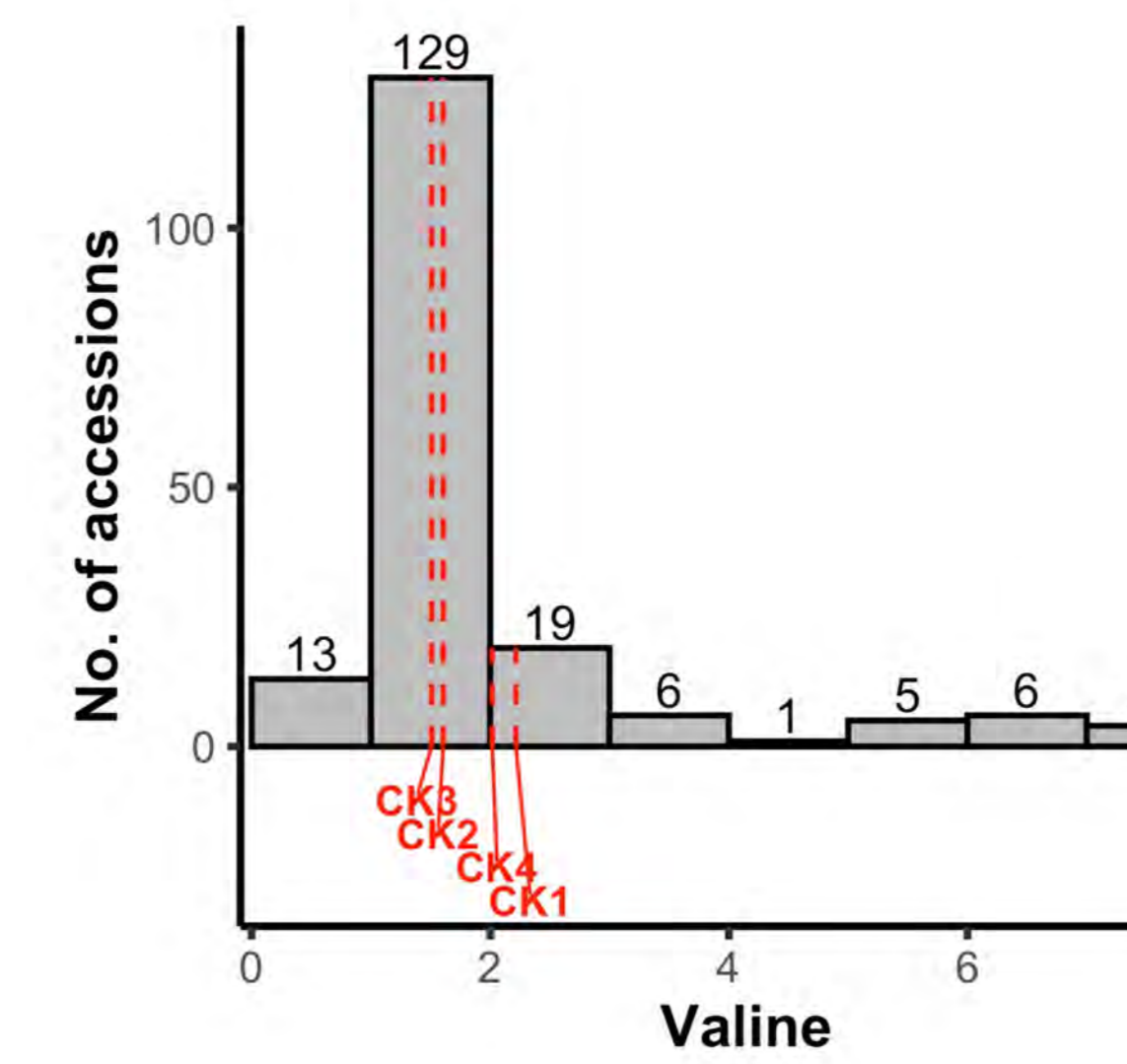

Supplement: jkag119_Supplementary_Data [file jkag119_supplementary_data.zip › Supplemental_Fig_2_G3-2026-406764.pdf]

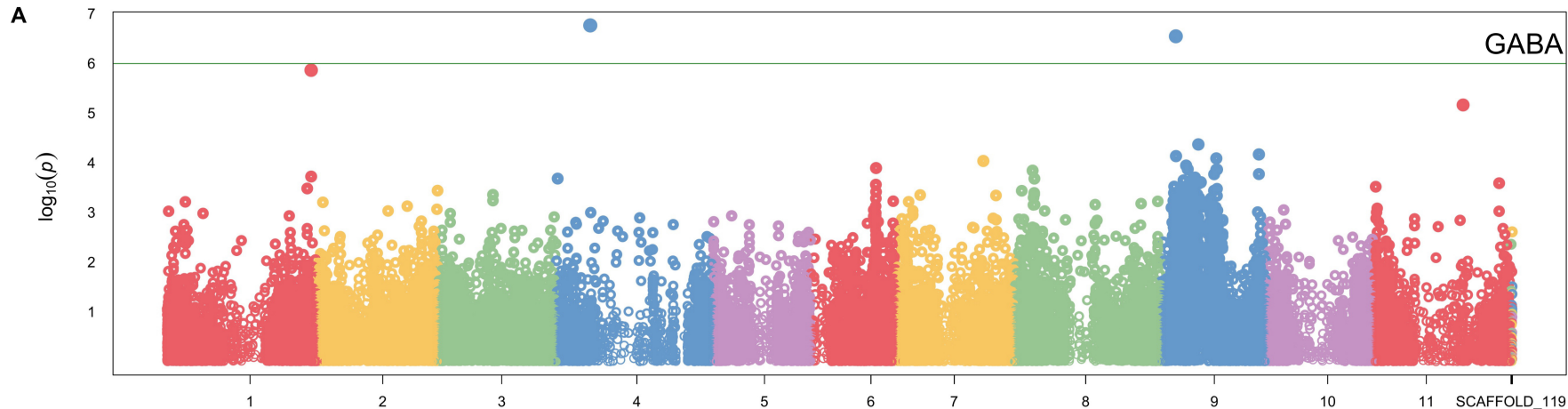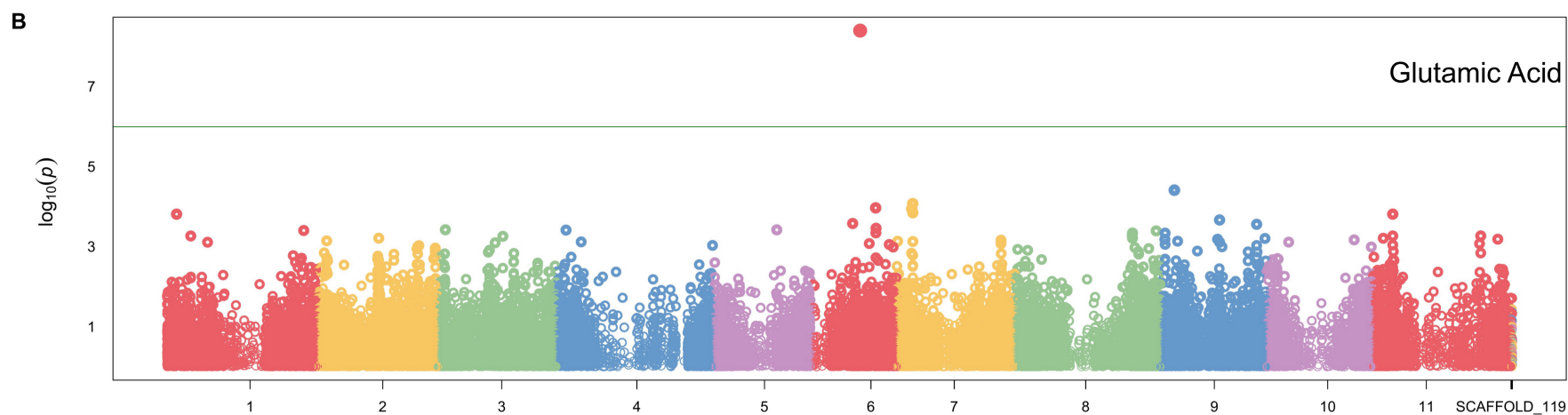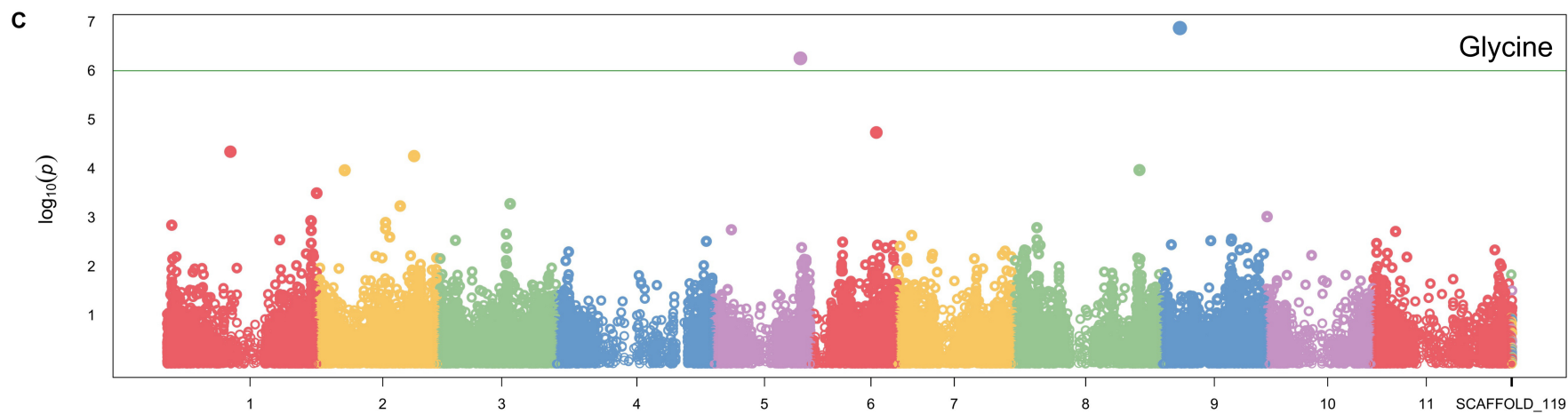

Supplement: jkag119_Supplementary_Data [file jkag119_supplementary_data.zip › Supplemental_Fig_3_G3-2026-406764.pdf]

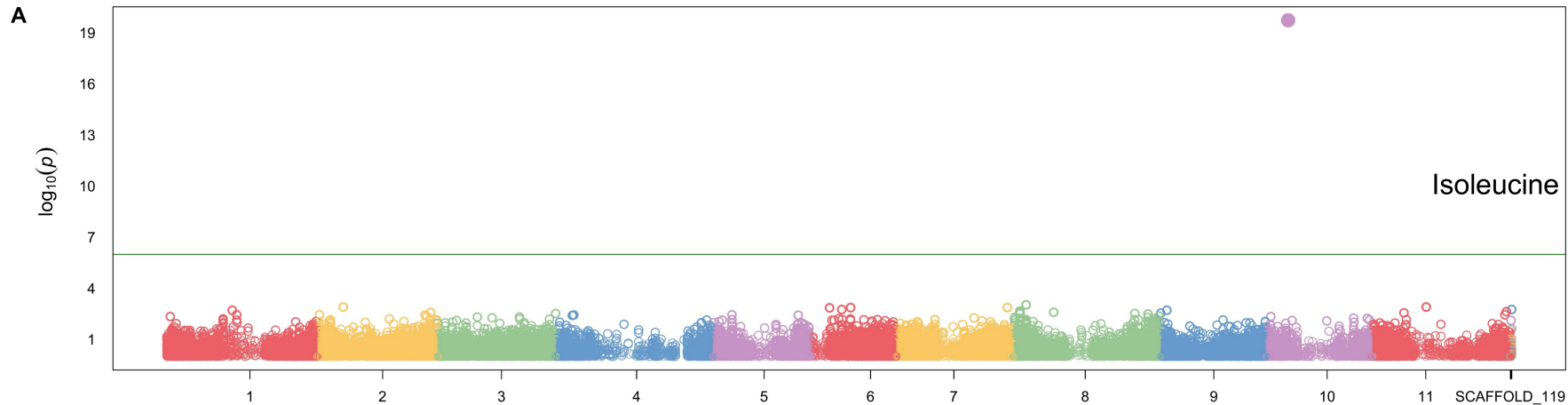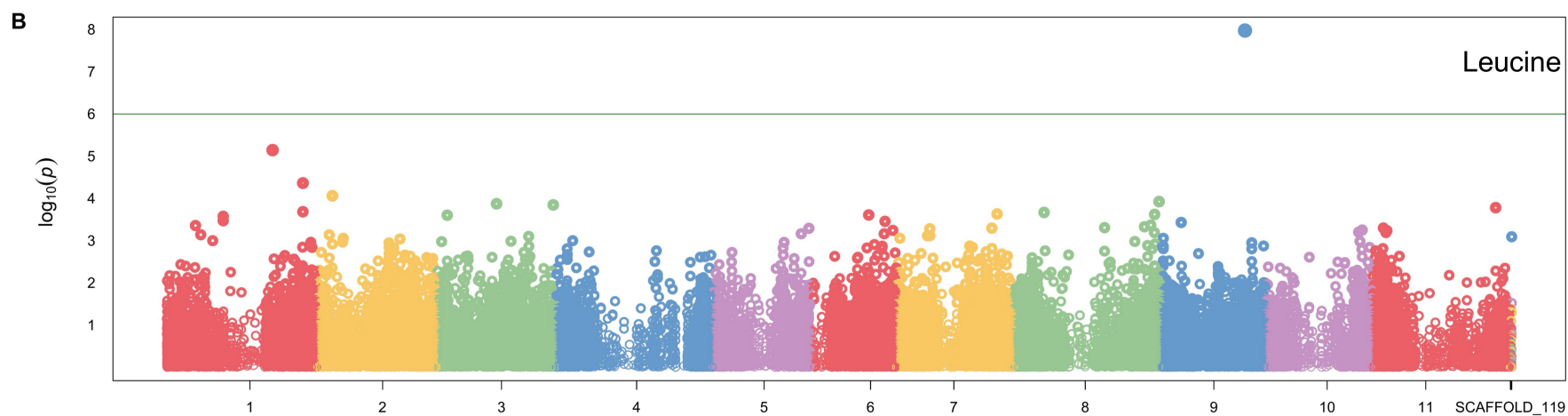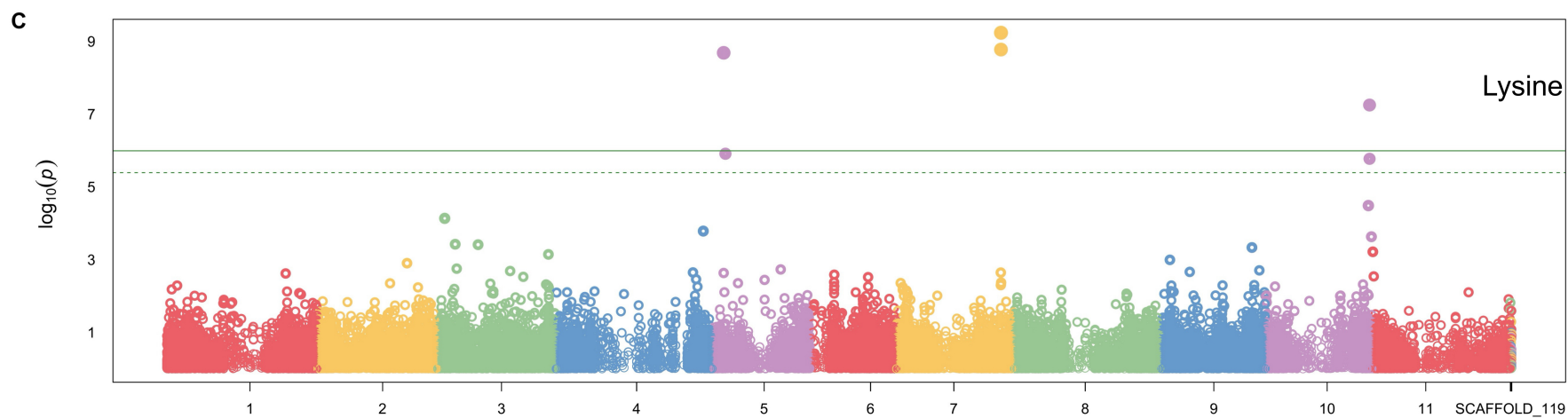

Supplement: jkag119_Supplementary_Data [file jkag119_supplementary_data.zip › Supplemental_Fig_4_G3-2026-406764.pdf]

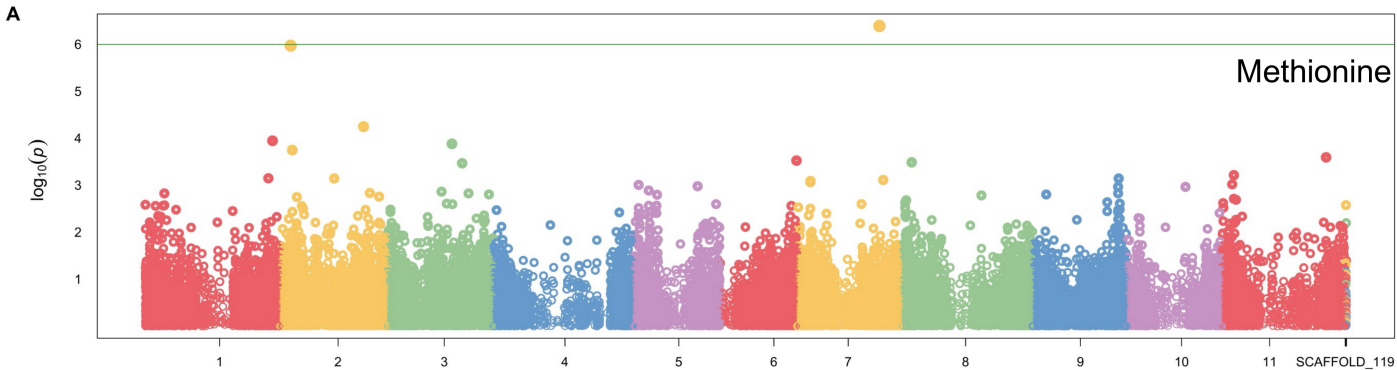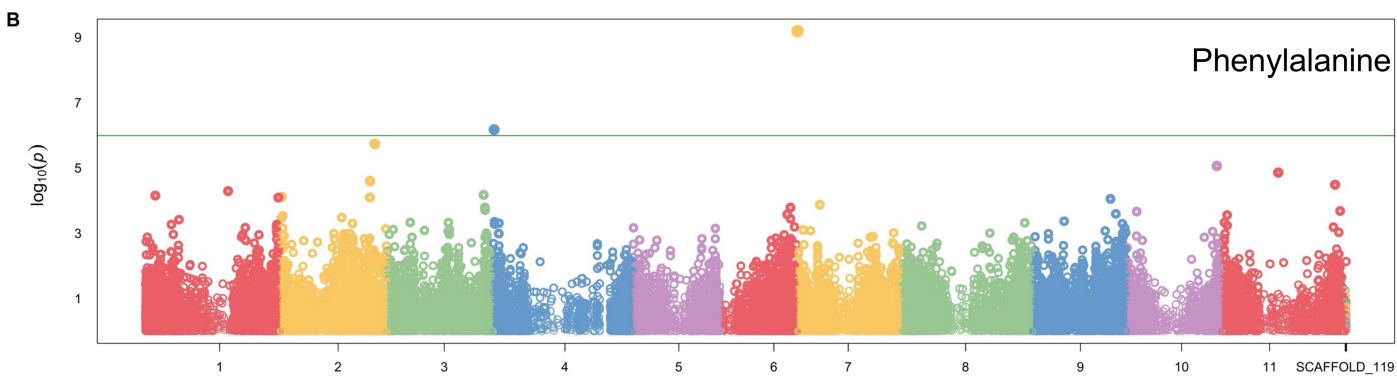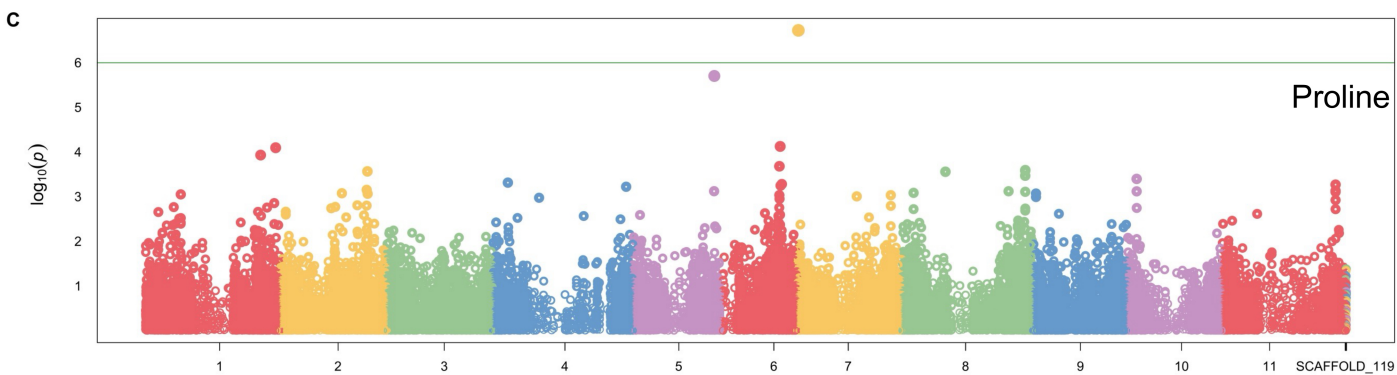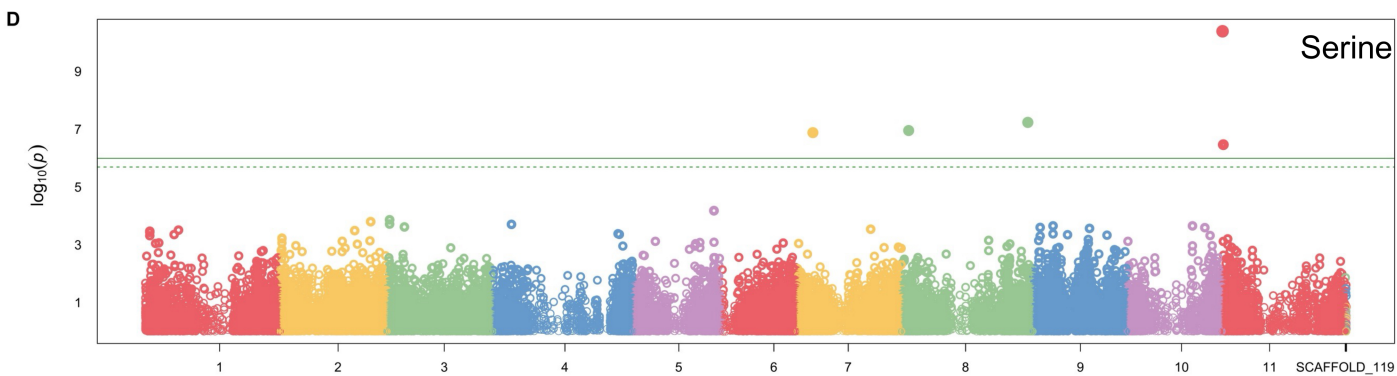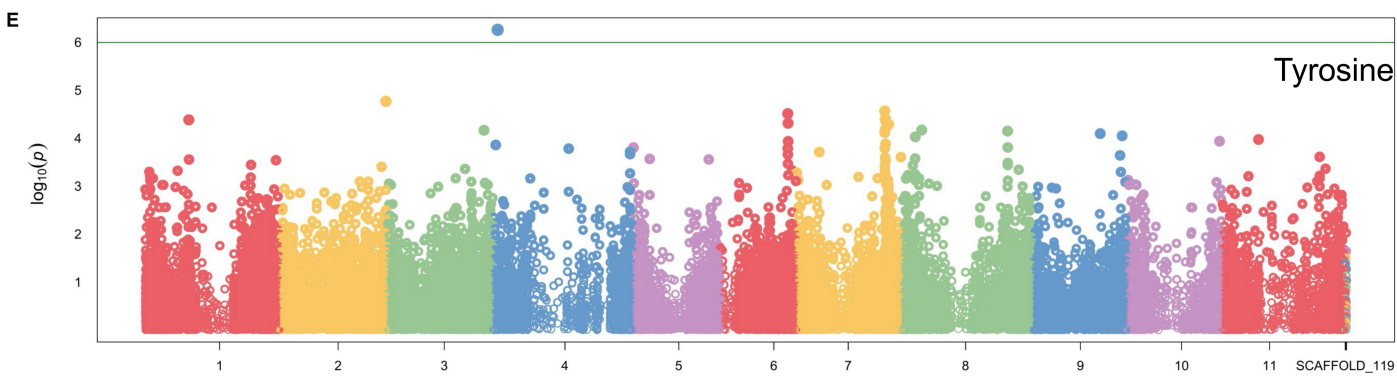

Supplement: jkag119_Supplementary_Data [file jkag119_supplementary_data.zip › Supplemental_Fig_5_G3-2026-406764.pdf]
